# Characterization of X-Chromosome Gene Expression in Bovine Blastocysts Derived by *In vitro* Fertilization and Somatic Cell Nuclear Transfer

**DOI:** 10.3389/fgene.2017.00042

**Published:** 2017-04-10

**Authors:** Byungkuk Min, Jung Sun Park, Kyuheum Jeon, Yong-Kook Kang

**Affiliations:** Development and Differentiation Research Center, Korea Research Institute of Bioscience BiotechnologyDaejeon, South Korea

**Keywords:** X chromosome reactivation (XCR), X chromosome inactivation (XCI), SCNT, RNA-seq, bovine embryos

## Abstract

To better understand X-chromosome reactivation (XCR) during early development, we analyzed transcriptomic data obtained from bovine male and female blastocysts derived by *in-vitro* fertilization (IVF) or somatic-cell nuclear transfer (SCNT). We found that X-linked genes were upregulated by almost two-fold in female compared with male IVF blastocysts. The upregulation of X-linked genes in female IVFs indicated a transcriptional dimorphism between the sexes, because the mean autosomal gene expression levels were relatively constant, regardless of sex. X-linked genes were expressed equivalently in the inner-cell mass and the trophectoderm parts of female blastocysts, indicating no imprinted inactivation of paternal X in the trophectoderm. All these features of X-linked gene expression observed in IVFs were also detected in SCNT blastocysts, although to a lesser extent. A heatmap of X-linked gene expression revealed that the initial resemblance of X-linked gene expression patterns between male and female donor cells turned sexually divergent in host SCNTs, ultimately resembling the patterns of male and female IVFs. Additionally, we found that sham SCNT blastocysts, which underwent the same nuclear-transfer procedures, but retained their embryonic genome, closely mimicked IVFs for X-linked gene expression, which indicated that the embryo manipulation procedure itself does not interfere with XCR in SCNT blastocysts. Our findings indicated that female SCNTs have less efficient XCR, suggesting that clonal reprogramming of X chromosomes is incomplete and occurs variably among blastocysts, and even among cells in a single blastocyst.

## Introduction

X-chromosome inactivation (XCI) has evolved in female mammals to compensate for sex-chromosome dosage differences by suppressing gene expression from one X chromosome, and to render all cells as functionally monosomic for the X chromosome, a process considered to be important for normal embryonic development (Monk and Harper, 1979; Penny et al., 1996). The current understanding of XCI during early embryogenesis largely originates from studies in mice, partially because XCI occurs during a very early developmental window when the embryo is accessible. In female mice, dosage compensation takes place within a continual cycle of XCI and reversal, also known as X-chromosome reactivation (XCR) (Lee and Bartolomei, 2013). Inactivation of the paternal X chromosome occurs progressively during the first days of post-fertilization cleavage until the morula stage (Okamoto and Heard, 2006; Kalantry et al., 2009; Namekawa et al., 2010). At the late blastocyst stage, XCR is observed within cells from the inner cell mass (ICM) that will form the embryo proper (i.e., two active X chromosomes are present in these cells), whereas cells of the trophectoderm (TE), which will form the placenta, maintain imprinted inactivation of the paternal X chromosome (Mak et al., 2004; Okamoto et al., 2004; Patrat et al., 2009). At the onset of gastrulation, embryonic lineage cells randomly undergo XCI again without a parent-of-origin bias, and once random X-inactivation is initiated, all the progeny cells maintain the same X-inactivation status (Lyon, 1961). In contrast to embryonic lineage cells, extra-embryonic cells (i.e., TE cells) maintain the silenced paternal X chromosome throughout embryogenesis. Meanwhile, XCR can be induced during reprogramming of differentiated cells toward pluripotency by nuclear transfer, cell fusion, or ectopic expression of reprogramming factors (for a review, see Pasque and Plath, 2015; Payer, 2016; Vallot et al., 2016). Recently, it was shown that during the early period of reprogramming in hybrid cells between human fibroblasts and mouse embryonic stem cells, human nuclei undergo a loss of *XIST* and XCI-associated histone marks from the inactive X chromosome to accomplish XCR, although some regions on the X chromosome are refractory to reprogramming (Cantone et al., 2016).

However, species-specific differences are found in the patterns of X-inactivation and reactivation in mammals. Early human and rabbit embryos have different XCI initiation strategies compared with mice (Okamoto et al., 2011; Deng et al., 2014). Furthermore, in these species, *XIST* was not imprinted, and both X chromosomes remained active in the ICM and TE of blastocysts. In addition, several studies found that the paternal X chromosome does not undergo imprinted X inactivation in human embryos and in extra-embryonic tissues (Skuse et al., 1997; Skuse, 2005; Moreira de Mello et al., 2010; Penaherrera et al., 2012; Tachibana et al., 2012). Similarly, XCI does not occur in bovine blastocysts. Early studies found upregulation of X-linked genes such as *XIAP, G6PD*, and *HPRT* in female bovine blastocysts, despite strong *XIST* expression (Gutierrez-Adan et al., 2000; Peippo et al., 2002; Wrenzycki et al., 2002; Morton et al., 2007). Allelic expression analysis of the X-linked polymorphic *MAOA* gene showed preferential inactivation of the paternal X chromosome in bovine fetal placentae at approximately 100 days of gestation (Xue et al., 2002); however, more definitive studies with earlier stage embryos have not been reported. A microarray study using pooled *in vitro*-produced bovine blastocysts of known sexes showed that X-linked transcripts were mostly upregulated (Bermejo-Alvarez et al., 2010), which indicated that XCI does not occur at the blastocyst stage. Studies on XCI in non-rodent mammalian species demonstrated that X inactivation in mice might not apply to other species; therefore, determining the similarities and differences in XCI among mammalian species is very important.

Global gene expression studies using bovine pre-implantation embryos are scarce, and investigations into X-inactivation/reactivation using transcriptomic data from early embryos are scarcer still. This is mostly because of the major technical barrier in dealing with a limited number of embryos in a minuscule volume. In this study, we generated RNA-seq data derived from *in vitro* bovine male and female blastocysts to identify and characterize gender-specific expression patterns of genes from the X chromosome and autosomes. To investigate differences between embryos of different origins, we included male and female somatic cell nuclear transfer (SCNT) embryos in the RNA-seq analysis. Furthermore, our RNA-seq data were obtained from single male and female blastocysts, which enabled analysis of individual blastocysts to investigate the variability in X chromosome gene expression profiles. Knowledge regarding XIC in non-rodent pre-implantation embryos is extremely limited; therefore, our study using bovine embryos will provide insights into the evolutionary and molecular aspects of X inactivation and reactivation that occurs in early mammalian embryos, including humans.

## Materials and methods

### Generation of bovine blastocyst samples and sexing

This study was carried out in strict accordance with the recommendations in the Guide for the Care and Use of Laboratory Animals of the National Livestock Research Institute of Korea. The protocol was approved by the Committee on the Ethics of Animal Experiments of the Korea Research Institute of Bioscience and Biotechnology.

The procedures for generation of bovine IVF and SCNT blastocysts were described in detail elsewhere (Kwon et al., 2015b). Briefly, single donor cell was injected into enucleated oocyte by a micromanipulator with an inverted microscope (Leitz), and each donor-oocyte complex was fused by an Electro Cell Manipulator 2001 (BTX). The fused eggs were activated 4 h after. Blastocysts were generated 6–8 days post-NT. The quality of each blastocyst was assessed by Hoechst staining, and only high quality embryos with 60–80 blastomeres were chosen for transcriptomic analysis. We used male and female bovine ear skin fibroblasts as donor cells which were passaged three times before SCNT.

For generation of sham SCNT blastocysts, 18–22 h post-IVF, the zygote with two parental pronuclei was chosen for manipulation (Park et al., 2007). To exactly mimic the physical damage of enucleation, zona pellucida was partially ripped and the polar body and a part of the underlying ooplasm were removed using a micropipette without touching either male or female pronucleus. After 2 h of incubation, the reconstructed oocytes were activated using 5 μM ionomycin (Sigma) for 5 min, followed by treatment with 2.5 mM 6-dimethyl-aminopurine (DMAP, Sigma) in CR1aa culture media supplemented with 0.3% BSA for 3.5 h. The oocytes were then *in vitro* cultured to the blastocyst stage.

Sexes of IVF or sham-SCNT blastocysts were determined by PCR with Y-specific primers (BY; 5′-CTCAGCAAAGCACACCAGAC-3′ and 5′-GAACTTTCAAGCAGCTGAGGC-3′) and bovine-specific primers (BSP; 5′-TTTACCTTAGAACAAACCGAGGCAC-3′ and 5′-TACGGAAAGGAAAGATGACCTGACC-3′) as previously reported (Rattanasuk et al., 2011). One-tenth volume of genomic DNA extracted from single blastocysts was amplified by PCR using AccuPower PCR PreMix (Bioneer) and PCR product was resolved on 2% agarose gel.

### Transcriptome amplification of single blastocysts by pico-profiling

Transcriptomic materials were extracted from total 35 blastocysts (6 male IVF, 6 male SCNT, 6 male sham, 6 female IVF, 6 female SCNT, 5 sham) and male/female donor cells and amplified by the pico-profiling method. The pico-profiling procedure was described in detail elsewhere (Min et al., 2015, 2016). Briefly, from each bovine blastocyst, poly-A tailed RNAs were extracted using Dynabeads mRNA DIRECT kit (Invitrogen) and reverse transcribed using 200 units of SuperScript III (Invitrogen). Pico-profiling was done using random primer harboring MlyI restriction enzyme site. The pico-profiled cDNA fragments were amplified using 5′ anchor primer by PCR with 20 cycles of 94°C for 2 min, 70°C for 5 min. Adapters were removed from amplicons by overnight digestion with MlyI restriction enzyme to produce double strand cDNA fragments suitable for Illumina NGS library generation.

### Preparation of NGS libraries

Illumina NGS libraries were generated using TruSeq DNA Sample Preparation kit (Illumina) according to the supplier's guide with several minor modifications. End-repair was performed using the whole pico-profiling amplicons (20 μl) by incubating with 25 μl End Repair Mix (Illumina) and 5 μl DW at 30°C for 2 h. The reactions were purified with AMPure XP beads (Beckman) and DNA was eluted in 12.5 μl DW. Then, 12.5 μl A-Tailing Mix (Illumina) and 5 μl DW were added into each sample, and the mixtures were incubated at 37°C for 2 h. Next, 1 μl barcoded adapter was added into each 3' end adenylated DNA sample with 2.5 μl Ligation Mix (Illumina) and 6.5 μl DW, and the mixtures was overnight incubated at 16°C. Ligates were purified twice using AMPure XP bead (Bechman) and eluted into 30 μl DW. For size selection, adapter ligated DNA samples were mixed with 10 μl loading solution (Sage Science) and loaded onto Pippin Prep (Sage Science). DNA samples were enriched by 18 cycles of PCR reaction comprising 5 μl size selected DNA fragments, 25 μl PCR Master Mix(Illumina), 1 μl PCR Primer Cocktail (Illumina), and 19 ul DW. Finally, enriched DNA fragments were purified and sequenced using HiSeq2500. Male and female samples were separately pooled and sequenced using two flow cells of HiSeq2500 system.

### Bioinformatic analyses

Raw reads from HiSeq2500 (100 bp, PE) were preprocessed using “trim_galore” to remove Illumina adapter sequences and low quality bases, and the trimmed reads were aligned on *Bos taurus* UMD3.1 (NCBI) with TopHat2 (Trapnell et al., 2012; Kim et al., 2013). We followed the Tuxedo suit pipeline (TopHat, Cufflinks, and CummeRbund) with default parameters for mapping, expression estimation and differential expression (DE) analysis (Trapnell et al., 2012). For the estimation of gene expression levels, Cufflinks with -G option was used to calculate the abundance of only known transcripts due to the incomplete genomic annotation of the bovine genome.

For sliding window analyses, each chromosome was binned into 1 megabase windows. FPKM values of genes in each window were summed up, and relative expression levels against mean expression levels of male IVF blastocysts were calculated. In order to plot the calculated relative expression, FPKM values were smoothened by averaging 30 windows moving along each chromosome. All plots in this study were generated using in-house R scripts, Origin (OriginLab), or Excel (Microsoft).

## Results

### Experimental scheme and validation of pico-profiling-sequencing method through a pilot experiment

**Figure 1:**
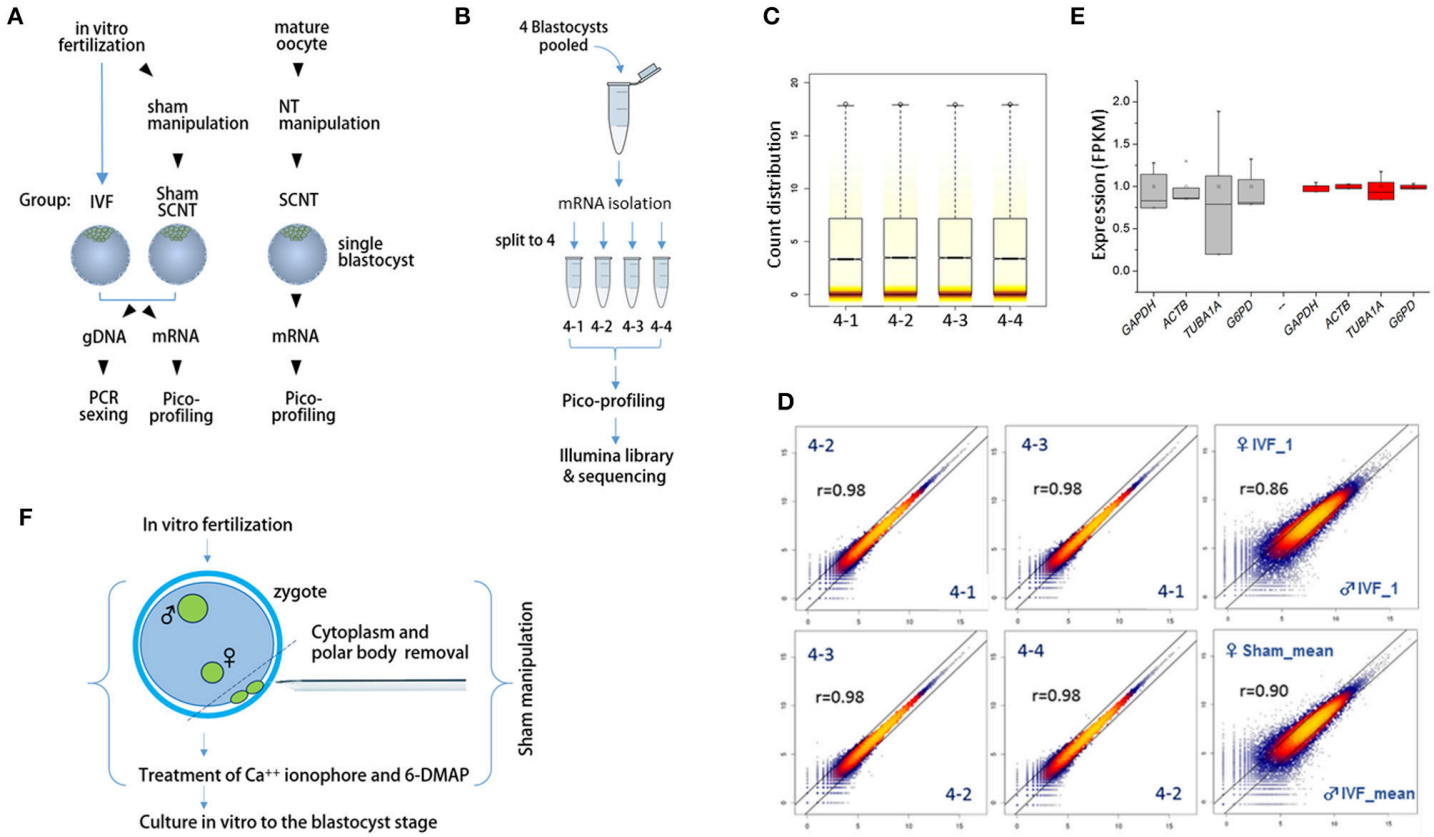
**Schematics of the sample preparation and pico-profiling processes for deep sequencing. (A)** Preparation of bovine blastocyst samples for sexing and pico-profiling. Blastocysts were derived by *in vitro*-fertilization (IVF) or somatic cell nuclear transfer (SCNT). Adult ear skin fibroblasts were used as donor cells to obtain SCNT embryos. Genomic DNA and mRNA were extracted from single IVF and sham nuclear transfer (NT) blastocysts for sexing and pico-profiling. **(B–E)** Validation of the pico-profiling method. In **(B)**, there is an overview of the experimental strategy for validation using four identical replicates of pooled blastocyst samples. In **(C)** and **(D)**, sequencing results are presented. Box plot in **(C)** shows the distribution of total gene expression levels [log_2_(FPKM)] for each replicate. Colored stripes indicate gene density within a specific expression level. Scatter plots in **(D)** show the correlation between replicates. For comparison, other non-technical replicates (i.e., male IVF and female IVF blastocyst samples) are included. Pearson correlation coefficients (r) are denoted on each plot. In E, expression levels of housekeeping genes are compared between replicate groups (red) and other independent sample groups (gray). Note significantly less variability in the expression levels of the replicates compared to that found in other sample groups. **(F)**, Generation of sham NT embryos. Sixteen to 18 h after IVF, a zygote with maternal and paternal pronuclei was manipulated to remove the polar bodies plus a portion of cytoplasm underneath them, followed by a standard nuclear transfer protocol to develop to the blastocyst stage.

We extracted mRNA and genomic DNA from single bovine IVF blastocysts (IVF-BLs) for pico-profiling of transcripts (Min et al., 2015, 2016) and gender identification, as illustrated in Figure 1A. Complementary DNA (cDNA) amplicons from six male and six female IVF-BLs were used for RNA-seq library construction. First, we evaluated the pico-profiling-sequencing (Pip-seq) method in a preliminary experiment, using total RNA from a pool of four IVF-BLs that was divided into four parts for Pip-seq library construction (Figure 1B). The sequencing results showed that the distributions of normalized counts in the four replicates were comparable (Figure 1C), and the correlation was higher among the replicates (*r* = 0.98) compared with those between other IVF-BLs (*r* = 0.86–0.90; Figure 1D). In addition, we found that the replicates had significantly less variable FPKM (fragments per kilobase of exon per million mapped fragments) values for housekeeping genes such as *GAPDH* and *ACTB* compared to those found in a separate RNA-seq dataset derived from single blastocysts (Figure 1E). These results demonstrated that Pip-seq is a reliable method of cDNA amplification for use with minute samples, such as individual preimplantation embryos. In addition to IVF-BLs, we included SCNT blastocysts (SCNT-BLs) in our analysis, which were generated from adult ear skin fibroblasts under standardized SCNT conditions (Kwon et al., 2015a). As a reference, we included sham nuclear transfer blastocysts (sham NT-BLs) that mimicked SCNT-BLs because they underwent the same nuclear transfer procedure except that they retained their intact genomic material (Figure 1F). From Pip-seq, we obtained 37 transcriptomes from 35 blastocysts and two donor cells (see Supplementary Table S3 for the details of samples and sequencing results). We found that *XIST* was expressed abundantly in female blastocysts, and male-specific genes such as *UTY, DDX3Y*, and *EIF2S3Y* were expressed exclusively in male blastocysts (Supplementary Figure S1).

### X-linked genes were upregulated in bovine female blastocysts

**Figure 2:**
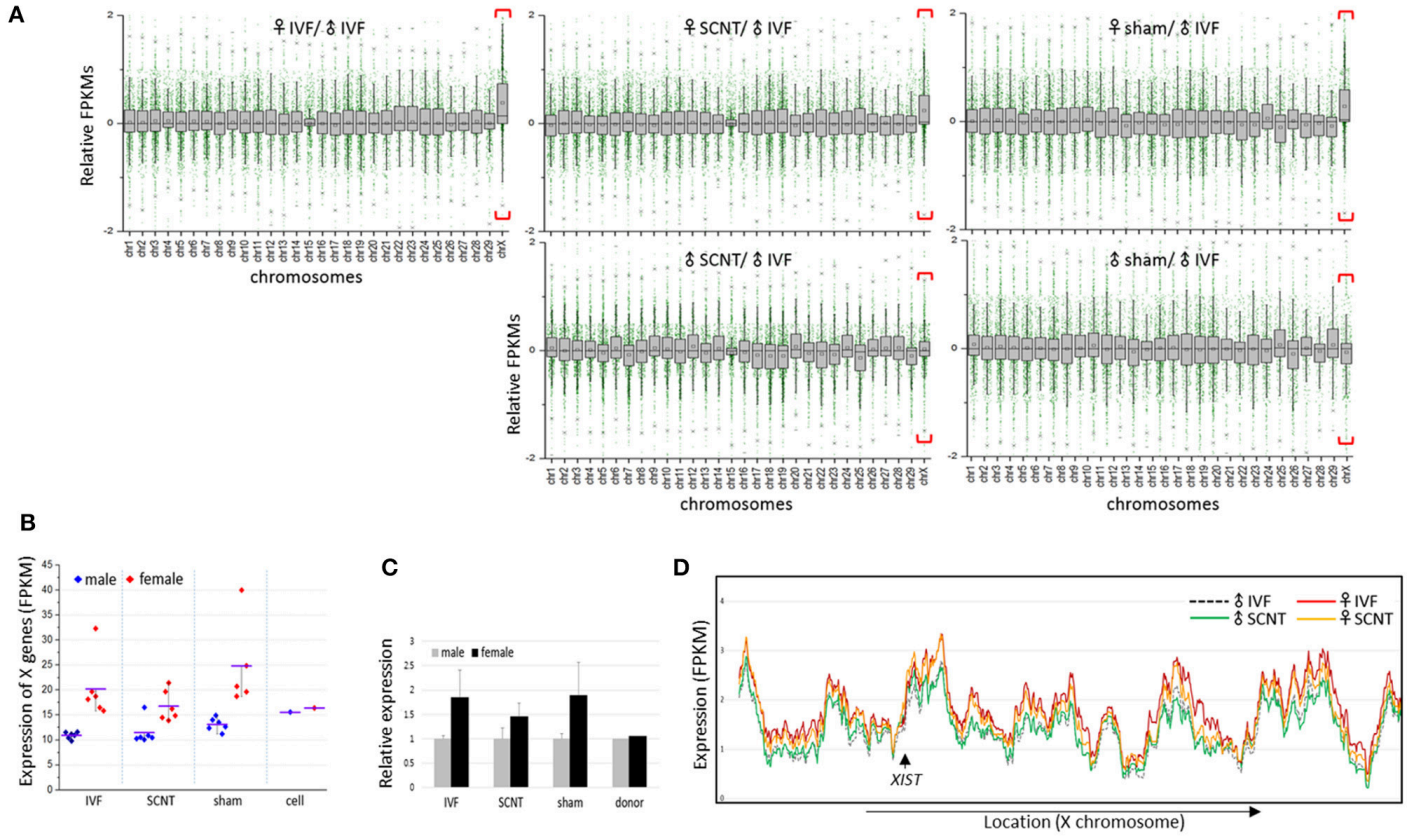
**Upregulation of X-chromosome genes in bovine female blastocysts. (A)** Box plots showing relative gene expression levels in FPKMs (fragments per kilobase of exon per million mapped fragments) of IVF, SCNT, and sham NT blastocysts plotted from chromosome 1 to X (excluding the Y chromosome) against the mean FPKM of male IVF blastocysts. X chromosomes are distinguished by higher mean expression levels (red brackets). Upper panel, females; lower panel, males. **(B)** Mean FPKMs of X-chromosome genes (X-linked genes) in individual embryos (dots) from IVF, SCNT, and sham NT groups. Donor cells (cell) are included. Blue, male; red, female; black line, group mean FPKM. **(C)**, Relative expression of X-linked genes in female blastocysts (black) compared to that in male blastocysts (gray). Error bars, standard deviation. **(D)** Comparison of chromosome-scale X-linked gene expression patterns using sliding window analysis between male and female samples, and between IVF and SCNT groups. Mean expression of X-linked genes in IVF and SCNT groups are plotted in order of genomic location. The location of the *XIST* gene is indicated (arrow).

We calculated the mean expression levels of individual genes in each group of blastocysts. Plotting the relative mean expression levels for each group to the mean expression levels for the male IVF group against chromosome, we found that X-linked genes were upregulated specifically in the female blastocyst groups (Figure 2A). In a comparison of the mean expression levels between male and female X-linked genes, we found a clear distinction between the sexes in each blastocyst group, in which female blastocysts had high and variable expression, whereas male blastocysts had relatively low and constant expression (Figure 2B). The mean female to male X-linked gene expression ratios were estimated as 1.8, 1.5, and 1.9 in the IVF, SCNT, and sham groups, respectively (Figure 2C). The almost two-fold higher abundance of X-linked gene transcripts indicated that both maternal and paternal X chromosomes are active in bovine female blastocysts, despite the presence of *XIST* transcripts, and suggested a late onset of XCI, similar to that observed in humans (Okamoto et al., 2004).

Plotting X-linked gene expression along the chromosome, we found that the expression patterns were very similar in all blastocyst groups (sham groups omitted), with an overall higher level of expression in the female groups, which indicated that upregulation of X-linked genes in female blastocysts does not occur locally, but is chromosome-wide (Figure 2D). Notably, female SCNT-BLs exhibited a lower profile compared to that found in female IVF-BLs, which is consistent with the lower FPKMs of X-linked genes found in female SCNT-BLs compared with that found in female IVF-BLs (Figure 2C). In addition, although the expression levels of X-linked genes differed between male and female SCNT-BLs, their expressions were very similar in male and female donor cells (Supplementary Figure S2), which suggested sexual differentiation in transcriptomes during SCNT development.

### The distribution of female-to-male ratios of X-linked genes was female-biased in both IVF-BLs and SCNT-BLs

**Figure 3:**
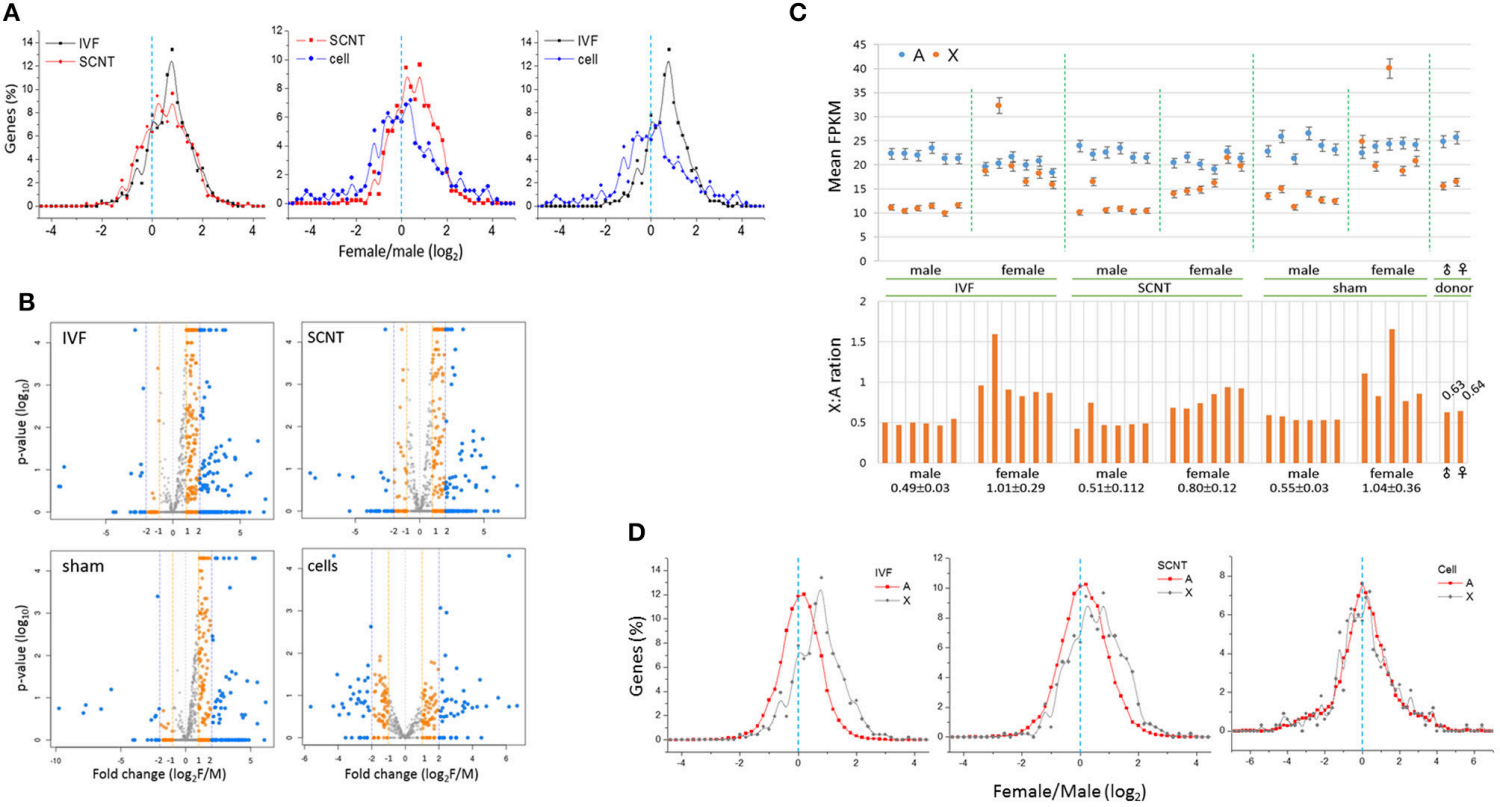
**Distribution of female-to-male ratios of X-chromosome gene expression levels in bovine blastocysts. (A)** Distribution of female-to-male (F:M) ratios of expression levels of X-chromosome genes (X-linked genes) in bovine IVF (black) and SCNT (red) blastocysts, and donor cells (blue). The dotted line (cyan) denotes an F:M ratio of 1 [log_2_(F:M) ratio = 0]. Bin size, 0.2 in log_2_(FPKM). **(B)** Volcano plots showing mean F:M levels of individual X-linked genes against *p*-values and fold changes in each blastocyst group. Genes with different F:M fold changes are denoted in different colors. **(C)** Comparison of expression (mean FPKM ± STD) in autosomal (A, blue) and X-chromosome genes (X, orange) between males and females in IVF, SCNT, and sham-NT blastocysts. The X:A ratio that denotes the FPKM level in each blastocyst is provided in the lower panel; the mean X:A ratios (± STD) of individual groups are denoted below. Donor cells are included for reference. **(D)** Distribution of female-to-male ratios in autosomal (A, black) and X-chromosome genes (X, red) in IVF and SCNT blastocysts. A dotted line (cyan) denotes an F:M ratio of 1 [log_2_(F:M) ratio = 0]. Bin size, 0.2 in log_2_(FPKM). Donor cell samples are included for reference.

Next, we examined the distribution of female-to-male (F:M) ratios of the expression levels of X-linked genes in IVF-BLs and SCNT-BLs (Figure 3A). Overall, we found that the distribution was female-biased in both groups. However, in contrast to the SCNT group, donor cell populations had a relatively balanced distribution, which suggested that the symmetric F:M distribution pattern of donor cells drifted to an asymmetric, female-biased pattern in SCNT-BLs, as in IVF-BLs. Non-parametric analysis using the Mann-Whitney (MW) test and the two-sample Kolmogov-Smirnov (KS) test showed that distributions of the F:M ratios of X-linked genes with FPKMs > 1 were significantly different in SCNT vs. donor cells, and in IVF vs. donor cells (both *p* < 0.05), but not between IVF vs. SCNT groups (MW test: *p* = 0.860).

The numbers of X-linked genes expressed in IVF-BLs, SCNT-BLs, sham NT-BLs, and donor cells were 668, 666, 628, and 479, respectively. We used volcano plots to display the mean F:M levels of individual X-linked genes in each blastocyst group (Figure 3B), which showed that the majority of X-linked genes were upregulated in female blastocysts. Notably, however, the SCNT group still had a considerable number of X-linked genes that were expressed at higher levels in males. Proportions of X-linked genes that were highly expressed (F:M > 1) in female blastocysts were 78.7, 66.7, 78.1, and 51.2%, and those with an F:M ratio > 2 were 46.7, 36.2, 33.1, and 24.2% in IVF-BLs, SCNT-BLs, sham NT-BLs, and donor cells, respectively.

### Expressions levels of autosomal genes are balanced between bovine female and male blastocysts

We compared the mean expression levels of X-linked genes and autosomal genes (A-genes) in individual blastocysts, and found that the expressions of A-genes were relatively constant in all blastocysts, irrespective of the different X-linked gene expressions observed between the sexes (Figure 3C). Furthermore, we found that the male IVF group had a mean X:A ratio of 0.49 compared with a mean of 1.01 found in the female IVF group, revealing a significant difference between the sexes (Wilcoxon signed rank test; *p* = 6.0e-09). We found X:A ratios of 0.51 and 0.80 in male and female SCNT-BLs, respectively, which were similar to those found in IVF-BLs, although slightly lower in female SCNT-BLs compared with those in female IVF-BLs.

We compared the distributions of F:M ratios of A-gene expression levels in IVF and SCNT blastocysts, and donor cell groups (Figure 3D). The F:M distribution was not skewed for A-genes in these groups, centering on an F:M ratio of 1 (log_2_ ratio = 0). Using an MW test, we found that the F:M distributions between X- and A-genes were significantly different between the IVF and SCNT groups (*p* < 0.002), but not in the donor cells (*p* > 0.05). In addition, examining autosomal differentially expressed genes (aDEGs; *p* < 0.05) between the sexes, we found that the number of male-high or female-high aDEGs was similar in both IVF (51.0 vs. 49.0%, respectively; *n* = 2,020) and SCNT (48.8 vs. 51.2%; *n* = 3,051) groups. This probably indicated that X chromosomes impose an extensive transcriptional regulation on A-genes in both IVF and SCNT blastocysts, as previously suggested (Bermejo-Alvarez et al., 2010).

### Features of X-linked differentially expressed genes and in the de-repression of X-linked genes

**Figure 4:**
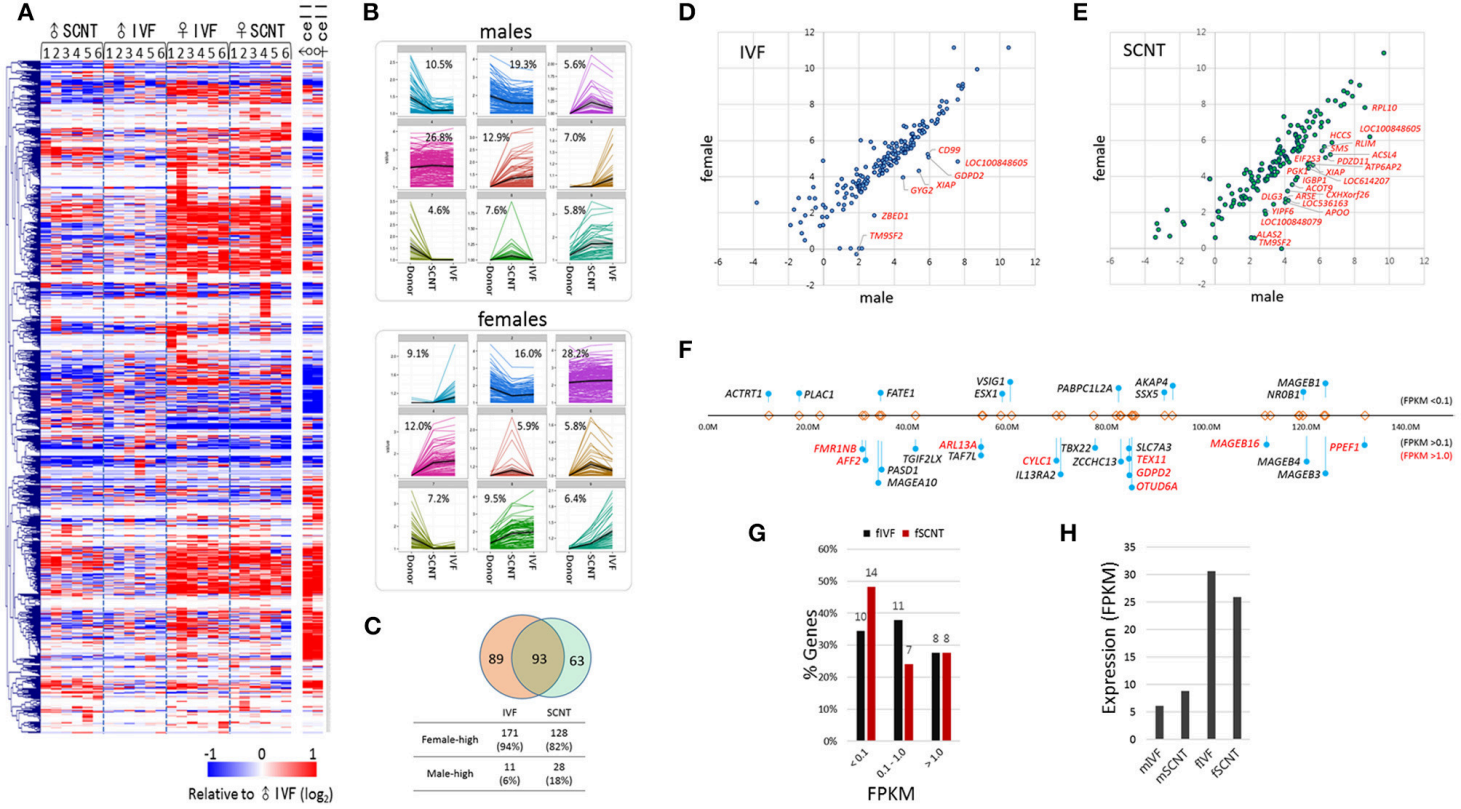
**Features of X-chromosome gene expression in IVF and SCNT blastocysts. (A)** Heatmap of relative X-chromosome gene (X-linked gene) expression in individual IVF and SCNT blastocysts vs. the mean expression level of the male IVF blastocyst group. Donor cells are included for reference. Genes are hierarchically clustered. **(B)** *k*-mean clustering (*k* = 9) of X-chromosome genes among donor cell, SCNT, and IVF blastocyst groups. The frequency of each cluster is denoted in the panel. Upper panel, males; lower panel, females. **(C)** Venn diagram showing the overlap between 182 IVF X-linked differentially expressed genes (xDEGs, *p* < 0.05; orange) and 156 SCNT xDEGs (green). **(D–E)** Scatter plots of xDEGs in IVF **(D)** and SCNT **(E)** blastocyst groups. Most xDEGs are highly expressed in females, and high-expressing xDEGs in males (male-high) are partly denoted with gene symbols. **(F–H)** Respective location **(F)** and expression **(G–H)** of 29 X-linked genes in bovine IVF and SCNT blastocysts specifically expressed in human and mouse testes. In **(F)** the relative positions and distances of genes on the X chromosome are indicated by “lollipops.” Genes are categorized by expression level where a FPKM < 0.1 indicates a none-to-weak level of expression, a FPKM > 0.1 indicates weak-to-moderate expression, and a FPKM > 0.5 (red) indicates moderate-to-high expression. Comparison of the expression of 29 testes-specific genes between female IVF and SCNT groups **(G)**, and between males and females **(H)**.

A heatmap of X-linked gene expression revealed a gender-specific pattern in blastocysts, with higher levels found in female blastocysts (Figure 4A). The heatmap pattern of female SCNT-BLs differed from that of male SCNT-BLs, and resembled that observed in female IVF-BLs. In addition, we found that male and female donor cells had similar X-linked gene expression patterns, but neither the pattern observed in female SCNT-BLs nor that found in male SCNT-BLs was similar to the pattern found in donor cells. Figure 4B shows a cluster analysis of X-linked gene expression, in which each cluster presents the pattern of change in gene expression between donor cells and SCNT-BLs, using the pattern seen in IVF-BLs as a reference. The genes that belong to each category are listed in the Supplementary Table S1.

Next, we detected and evaluated differentially expressed X-linked genes (xDEGs; *p* < 0.05) between the sexes (Figure 4C). In the IVF group, we found 182 xDEGs, of which 94% (171/182) were highly expressed in female blastocysts (Supplementary Table S2). We found that only a small proportion (6%) of xDEGs, including *GYG2, CD99, GDPD2, ZBED1, XIAP, TM9SF2*, and LOC100848605 were highly expressed in male IVFs (Figure 4D). Of these genes, *ZBED1* and *CD99* are located in the pseudoautosomal region 1 (PAR1) and therefore, are present on both the X and Y chromosomes. We propose that this male-biased expression resulted from partial spreading of XCI in females (Johnston et al., 2008). Furthermore, *GYG2* is known to have a short truncated version on the Y chromosome in humans (Zhai et al., 2000).

By contrast, in the SCNT group, we found 156 xDEGs (Supplementary Table S2), of which 18% (28/156) were highly expressed in males (Figure 4E). This proportion was larger than that observed in the IVF group, and it would interesting to determine whether these genes reside at loci that are refractory to X-chromosome reprogramming. We found that 93 xDEGs were common to IVF-BLs and SCNT-BLs (Figure 4C), and among them, *XIAP, TM9SF2*, and LOC100848605 were identified to be male-high. The regulatory mechanism involved in the expression of these male-high X-linked genes is currently unknown.

Certain male-specific X-linked genes are expressed specifically in the testes, the majority of which are expressed predominantly or exclusively at both pre- and post-meiotic stages (Wang et al., 2001; Mueller et al., 2008). Using previously reported data (Wang et al., 2008) as a reference, we selected 68 X-linked genes that have testes-specific expression in humans and mice, and identified annotations for 29 of these genes in the bovine genome browser (Figure 4F). We found that these genes are not localized at specific loci or regions, but are distributed over the entire X chromosome. In our blastocyst samples, we found that more than half of these 29 genes were expressed at an FPKM > 0.1 (Figure 4G), and that their expression levels were 3–5 times higher than that found in female blastocysts (Figure 4H). Therefore, our findings indicated that upregulation of the X chromosome or XCR leads to a de-repression of a large number of X-linked genes, including testes-specific genes, which are not necessary at the blastocyst stage. Furthermore, the data suggest a randomness and non-selectiveness in the de-repression of X-linked genes during XCR.

### X chromosome genes were similarly expressed in inner cell mass cells and trophectoderm cells

**Figure 5:**
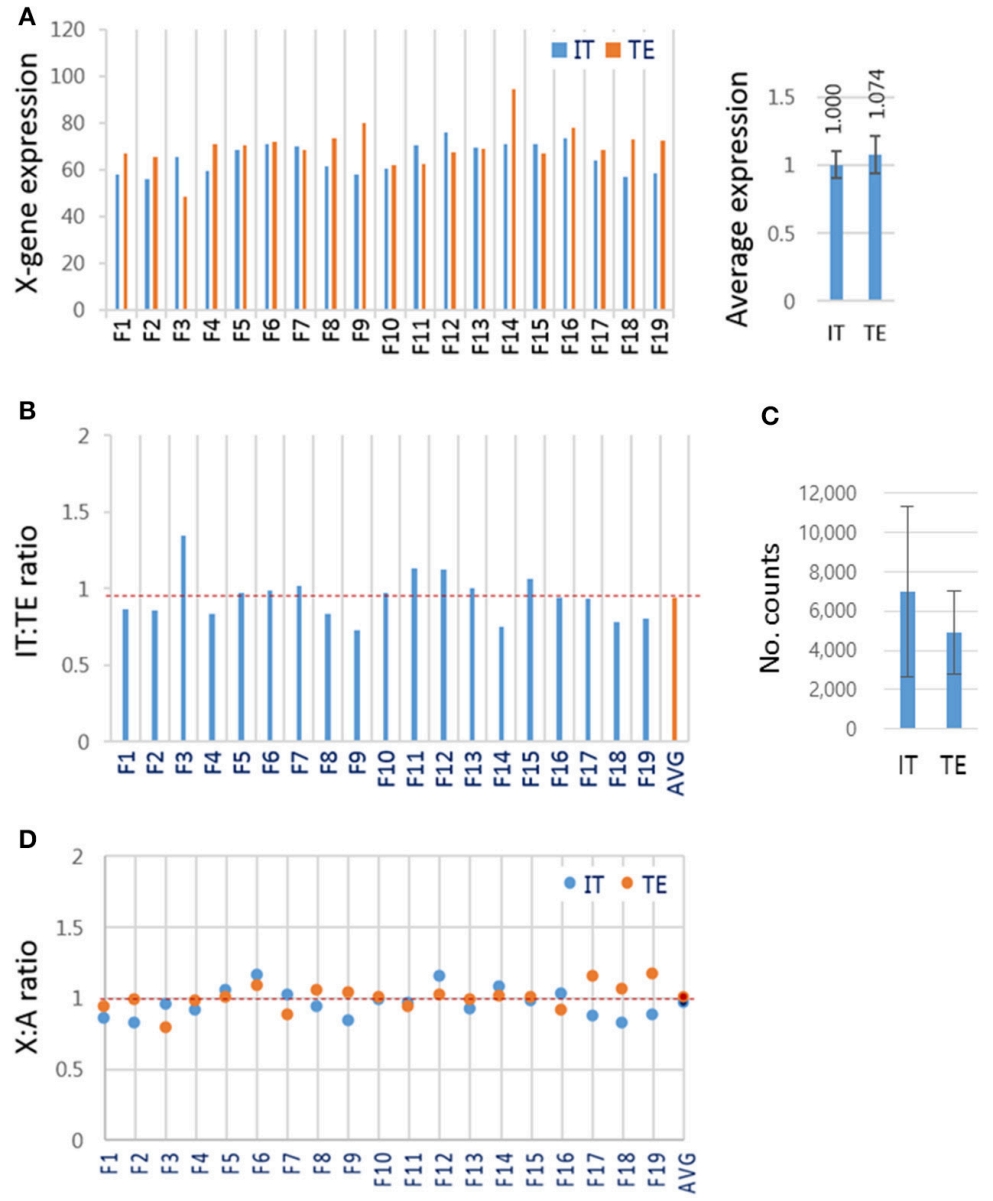
**X-chromosome gene expression in inner cell mass and trophectoderm lineage cells of female IVF blastocysts. (A)** The mean expression levels of X-linked genes in different lineage cells of the blastocyst. Female IVF blastocyst was physically split using a syringe needle into the “IT” part containing both ICM and TE cells and the TE-only part and separately subjected to RNA-seq. Right panel, the mean expression levels of X-linked genes in the IT and TE cells. **(B)** The relative expression of X-linked genes in IT to that in TE part in each blastocyst. **(C)** The mean *XIST* expression levels in read counts. Error bars, standard deviations. **(D)** The mean X:A ratios in IT (blue) and TE (red) cells. AVG, average IT/TE ratio (see also dotted red line).

Blastocysts contain cells of two different lineages, the ICM cells and trophectoderm (TE) cells. These two groups of cells might, as in mouse blastocyst (Mak et al., 2004; Okamoto et al., 2004; Patrat et al., 2009), have different strategies to express X-linked genes: ICM cells having both maternal and paternal active X chromosomes, and TE cells having active maternal and inactive paternal X chromosomes. To test whether this was the case for bovine blastocysts, female IVF blastocysts (*n* = 19) were dissected physically into two parts, the ‘IT’, containing both ICM and TE cells (almost 1:1 ratio in cell number), and the TE-only part, and subjected to RNA-seq separately. We found that the mean expression levels of X-linked genes were similar in the IT and TE cells (1.000 ± 0.098 and 1.074 ± 0.137, respectively; Figure 5A). If the paternal X had been imprinted in the TE part, the expression level of X-linked genes would have been higher in the IT part than in the TE part. The similarity in X-linked gene expressions between the IT and TE cells argued against the imprinted inactivation of paternal X. The relative expressions of X-linked genes in the IT to those in the TE in respective blastocysts were 0.945 on average, ranging from 0.80 to 1.18 (Figure 5B). There was no difference in the expression level of *XIST* at *p* < 0.01 level between the IT and TE parts (Figure 5C), which represented further evidence against the imprinted X inactivation in TE cells. We compared the mean expression level of X-linked genes and A-genes in each part, and found that the mean X:A ratios were not different between IT and TE samples (0.967 ± 0.102 and 1.008 ± 0.089, respectively; *p* = 0.227; Figure 5D), and similar to the mean X:A ratio of whole female IVF blastocysts (1.01 ± 0.29) (Figure 3C). Thus, we failed to find evidence for imprinted inactivation of paternal X chromosome or a preferential inactivation of either of the parental X chromosomes in TE lineage cells; therefore, we interpreted the result as indicating that both maternal and parental X chromosomes are regulated similarly in ICM and TE lineage cells in bovine blastocysts.

## Discussion

We found that the expression of X-linked genes in female IVF-BLs were almost twice that found in male IVF-BLs (Figure 2C). In addition to differences in the global expression levels of X-linked genes, the respective expression patterns were different in some clusters between female and male IVF-BLs, as evident from the heatmap (Figure 4A). We interpreted these findings as being caused by differences in X-chromosome dosage between male and female blastocysts, which implied that there is no mechanism for dosage compensation in bovine blastocysts. Our interpretation is in agreement with the observation that the lack of dosage compensation for the most highly expressed X-linked genes is tolerated not only in early mouse embryos (Namekawa et al., 2010), but also in embryonic stem cells (Elling et al., 2011; Leeb and Wutz, 2011). The resulting chromosome copy-number difference results in proteomic and metabolomic differences between the sexes (Gardner et al., 2010), which renders pre-implantation embryos of either sex more sensitive (or conversely, resistant) to essential signals for survival from the reproductive tract and also to numerous environmental factors, such as population density, famine, season, and stress, which are known to influence the sex ratio in mammals (Kruuk et al., 1999; Zorn et al., 2002; James, 2009). As shown in the heatmap of X-linked gene expression, SCNT-BLs displayed a contrasting sexual dimorphism, as did IVF-BLs, which demonstrated that the transcriptomic differences among X-linked genes is significantly more prominent between the sexes than between blastocyst groups of different origins (e.g., IVF vs. SCNT). Therefore, if the bovine blastocyst has an innate gender-specific response or behavior in the reproductive tract during the peri-implantation period, the response of SCNT-BLs may not differ from that found in gender-matched IVF-BLs.

In addition, we found that the imprinted X chromosome derived from donor cells was reactivated in female SCNT-BLs. An important observation was that, possibly due to insufficient XCR, the measured values of X-linked gene expression in SCNT-BLs consistently sat between the respective measures of donor cells and IVF-BLs: (1) the mean FPKMs of X-linked genes (1.0, 1.5, and 1.8 in female donor cells, female SCNT-BLs, and female IVF-BLs, respectively; Figure 2C); (2) the proportion of X-linked genes having an F:M ratio > 1 (51.2, 66.7, and 78.7%, respectively; Figure 3B); (3) the intermediate profile for X-linked gene expression levels across the chromosome observed using a sliding window analysis (Figure 2D); and (4) the mean FPKMs of X-linked genes relative to A-genes (0.64, 0.80, and 1.01, respectively; Figure 3C). We propose that this “in-between” ranking reflects the existence of chromosomal domains, or certain cell lineages, which are resistant to XCR in female SCNT-BLs. An alternative explanation, which is mutually non-exclusive to the theory of XCR-resistant domains, is that female SCNT-BLs might comprise a mosaic of cells that have different X-chromosome states; for example, one active and one inactive X chromosome (XaXi), such as that found in human pluripotent stem cell populations (Silva et al., 2008; Dvash et al., 2010; Anguera et al., 2012). Whatever the reason for the observed intermediate expression of X-linked genes in female SCNT-BLs, our findings indicated that reprogramming is incomplete at the mid-blastocyst stage, and might occur variably among blastocysts and even among cells in a blastocyst.

Owing to XCR in SCNT embryos, the imprinted X chromosome that is inherited from the female donor cell becomes the active X chromosome. This occurs in female SCNT-BLs, but not in male SCNT-BLs. XCR may be another process that facilitates reprogramming of the X chromosome, which might be used by female SCNT embryos because of the exceptional reconfiguration of the imprinted X chromosome. Conversely, XCR might be a burden to female SCNT embryos, which causes a serious delay in other concurrent and linked events of reprogramming. As shown by the X-linked gene expression profiles (Figure 4A), XCR might differentiate female SCNT-BLs from male SCNT-BLs, which would inevitably establish a sexual dimorphism in male and female SCNT-BLs, and may affect their cloning efficiency. However, because sham NT-BLs were comparable to their IVF counterparts, we concluded that manipulation and other nuclear transfer procedures do not interfere with the XCR process in female SCNT-BLs.

We found that upregulation of X-linked genes does not entail upregulation of A-genes in female blastocysts. If it had been true, the distribution of the F:M ratios of A-genes would have been skewed toward females, similar to that found for X-linked genes. However, we found that all three blastocyst groups, which derived differently, exhibited a well-balanced F:M ratio for A-gene expression (Figure 3D). We detected 2,020 aDEGs from the comparison of male and female IVF blastocysts. These aDEGs were well-balanced in number between the sexes, forming 7% (1030 male-high and 990 male-high aDEGs) of ~14,300 transcripts expressed (FPKM > 0.1) in IVF blastocysts. This result differs from a previous result reporting an array-based analysis of global gene expression in bovine IVF blastocysts (Bermejo-Alvarez et al., 2010). In that study, out of ~9300 transcripts expressed in bovine blastocysts, 12% (~1100) were detected as female-high aDEGs and 17% (~1600) as male-high aDEGs; this difference appeared large enough to shift the A-gene F:M ratio off-center. Moreover, when the lists of DEGs was compared with our DEGs, only 42 out of 99 annotated DEGs (~42%) overlapped. The discrepancy could be explained by a report that the RNA-seq and microarray platforms can yield different DEG results (Zhang et al., 2015). In addition, there may be other explanations for the discrepancy; for example, different sample sizes, the need for cDNA amplification, sperm-sorting-based embryo sexing, and advanced data analytical tools. The limited availability of genomic materials for deep sequencing means that the study of the transcriptome of mammalian preimplantation-stage embryos remains under-investigated. Continued research effort will provide sufficient embryo transcriptome data for verification. In addition, the early embryo-specific Pip-seq method, by lowering significantly the current technical constraints that hamper transcriptomic analysis of early mammalian embryos, will likely have a role in these future studies.

## Author contributions

YK led the project and supervised the study. BM and YK designed the experiments and interpreted the results. JP provided IVF and SCNT embryos. KJ pico-profiled blastocyst samples. BM and YK jointly performed bioinformatic analyses. BM and YK wrote the paper. All authors read and approved the final manuscript.

### Conflict of interest statement

The authors declare that the research was conducted in the absence of any commercial or financial relationships that could be construed as a potential conflict of interest. The reviewer MC and handling Editor declared their shared affiliation, and the handling Editor states that the process nevertheless met the standards of a fair and objective review.

## Supplementary material

Supplementary Figure S1 **X- or Y-linked gene expression patterns**.

Supplementary Figure S2 **X-chromosome wide gene expression pattern in donor cells**.

Supplementary Table S1 **k-mean clustering of X-genes**.

Supplementary Table S2 **xDEGs between male vs. female blastocysts**.

Supplementary Table S3 **Sequencing depth and mapping rates**.
