# Supplementary figures and images for: Characterization of X-Chromosome Gene Expression in Bovine Blastocysts Derived by In vitro Fertilization and Somatic Cell Nuclear Transfer
Source: Front Genet. 2017 Apr 10;8:42. doi: 10.3389/fgene.2017.00042 (PMC5385346; doi:10.3389/fgene.2017.00042)

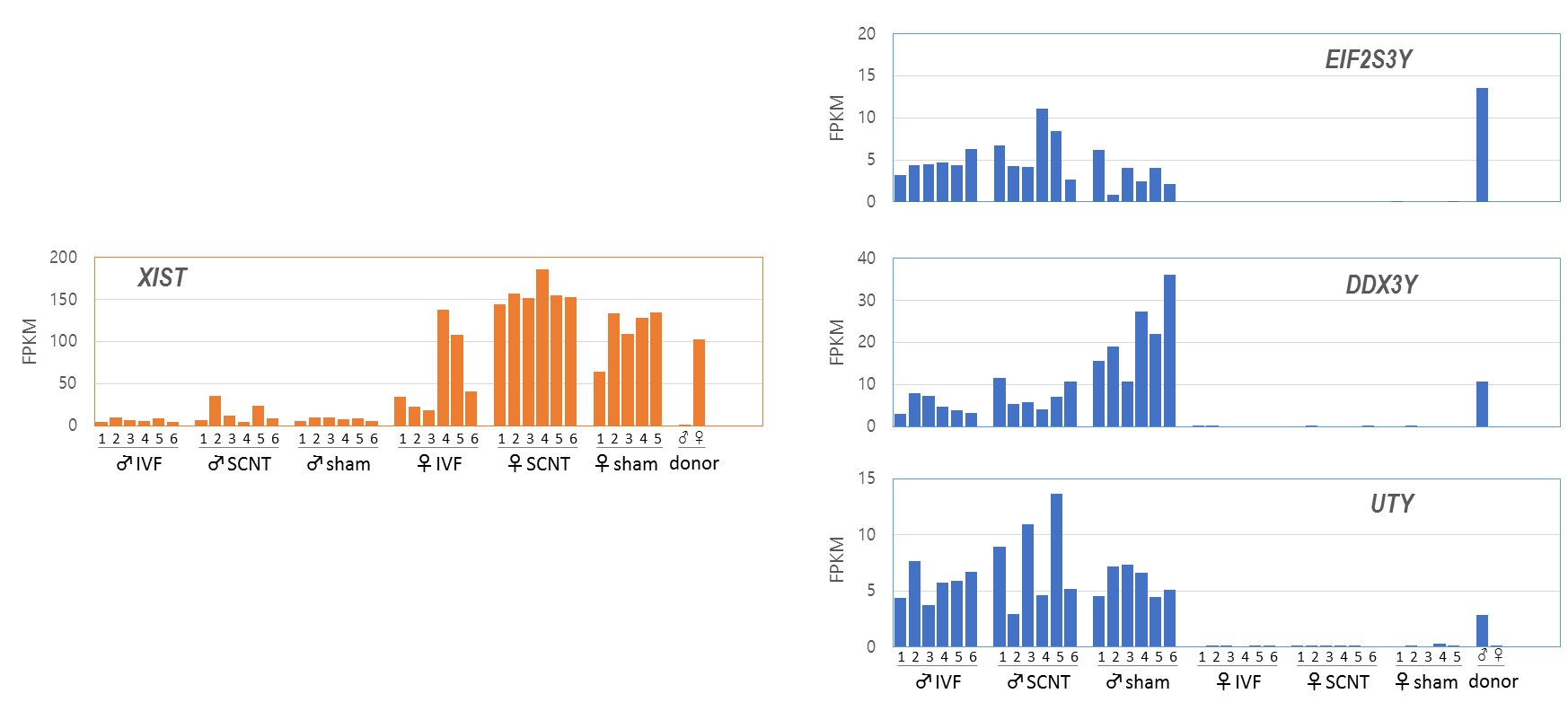

Supplement: Supplementary Figure S1 — X- or Y-linked gene expression patterns. [file Image1.TIF]

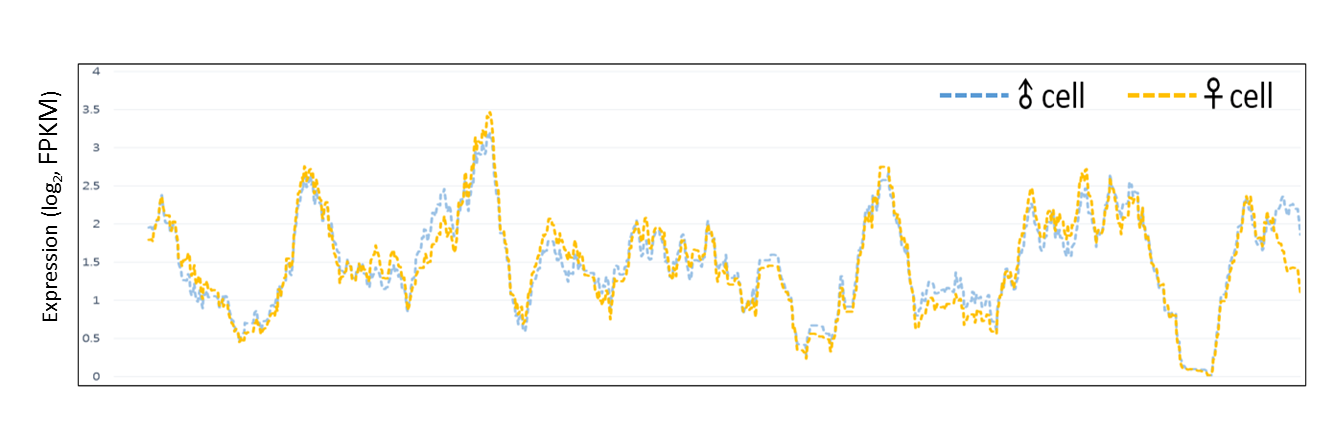

Supplement: Supplementary Figure S2 — X-chromosome wide gene expression pattern in donor cells. [file Image2.TIF]
