# Supplementary material for: Characterization of X-Chromosome Gene Expression in Bovine Blastocysts Derived by In vitro Fertilization and Somatic Cell Nuclear Transfer
Source: Front Genet. 2017 Apr 10;8:42. doi: 10.3389/fgene.2017.00042 (PMC5385346; doi:10.3389/fgene.2017.00042)
Supplement: Supplementary Table S1 — k-mean clustering of X-genes. [file Table1.PDF]

Table S1. k-mean clustering of X-genes

Symbol	cuff_ID	Cluster #
AGTR2	XLOC_021630	1
AKAP4	XLOC_022452	1
AP1S2	XLOC_022067	1
AR	XLOC_022416	1
ARMCX2	XLOC_022300	1
ARMCX3	XLOC_021816	1
ARSH	XLOC_022594	1
ASB12	XLOC_021992	1
ATP2B3	XLOC_021768	1
BRS3	XLOC_021703	1
CD99L2	XLOC_022202	1
CHIC1	XLOC_022384	1
CLCN4	XLOC_022610	1
CXHXorf38	XLOC_022011	1
CXHXorf57	XLOC_022309	1
DHRSX	XLOC_022084	1
EFNB1	XLOC_022413	1
F9	XLOC_021710	1
FIGF	XLOC_022070	1
FOXR2	XLOC_021982	1
GPC4	XLOC_022144	1
GPR173	XLOC_021971	1
GSPT2	XLOC_022458	1
HDX	XLOC_021873	1
HEPH	XLOC_022482	1
HIGD1A	XLOC_021869	1
HS6ST2	XLOC_022142	1
IL13RA2	XLOC_022179	1
IL1RAPL2	XLOC_021801	1
IQSEC2	XLOC_022464	1
ITGB1BP2	XLOC_022395	1
KIAA2022	XLOC_021884	1
LAGE3	XLOC_021785	1
LOC100302527	XLOC_021690	1
LOC100335556	XLOC_022365	1
LOC100336550	XLOC_021673	1
LOC100336959	XLOC_022323	1
LOC100847200	XLOC_022266	1
LOC100847415	XLOC_022161	1
LOC100847628	XLOC_022094	1
LOC100848281	XLOC_022393	1
LOC100848413	XLOC_022035	1
LOC507696	XLOC_021794	1
LOC518106	XLOC_021766	1
LOC540188	XLOC_021790	1

Symbol	cuff_ID	Cluster #
LOC540312	XLOC_021698	1
LOC615809	XLOC_021691	1
LOC782048	XLOC_022593	1
LOC787709	XLOC_022122	1
LOC788191	XLOC_022282	1
LRCH2	XLOC_022357	1
LRCH2	XLOC_022360	1
MGC134179	XLOC_021788	1
MID1IP1	XLOC_022498	1
MUM1L1	XLOC_021804	1
NDP	XLOC_022006	1
NHSL2	XLOC_022389	1
NLGN4Y	XLOC_022095	1
ODZ1	XLOC_022117	1
OPHN1	XLOC_021911	1
OTC	XLOC_022500	1
PIR	XLOC_022069	1
PLS3	XLOC_021863	1
PLXNA3	XLOC_022270	1
PRRG1	XLOC_022018	1
PTCHD1	XLOC_022562	1
RAB33A	XLOC_021670	1
RRAGB	XLOC_021983	1
S100G	XLOC_022577	1
SCML2	XLOC_022061	1
SHROOM4	XLOC_022455	1
SLC25A14	XLOC_021671	1
SLC38A5	XLOC_022432	1
SMARCA1	XLOC_022125	1
SPIN2	XLOC_022479	1
TBC1D8B	XLOC_022307	1
TCEAL1	XLOC_021824	1
TCEANC	XLOC_022587	1
WDR13	XLOC_021933	1
ZDHHC15	XLOC_021882	1
ZNF275	XLOC_021765	1
ZNF711	XLOC_022363	1
	XLOC_022420	1
ABCB7	XLOC_021883	2
ACRC	XLOC_022390	2
AIFM1	XLOC_022129	2
ALG13	XLOC_021849	2
APOOL	XLOC_022364	2
ARHGAP4	XLOC_022259	2
ARHGEF6	XLOC_022172	2
ARMCX4	XLOC_021813	2
ARR3	XLOC_022404	2
ARSD	XLOC_022082	2
ATG4A	XLOC_021841	2
ATP6AP2	XLOC_022497	2

Symbol	cuff_ID	Cluster #
ATP7A	XLOC_022374	2
BCORL1	XLOC_021668	2
BEX2	XLOC_022310	2
BEX5	XLOC_022303	2
BHLHB9	XLOC_021795	2
BRCC3	XLOC_022239	2
C1GALT1C1	XLOC_021651	2
CA5B	XLOC_022581	2
CD99	XLOC_022598	2
CETN2	XLOC_022213	2
CFP	XLOC_022424	2
CHM	XLOC_021866	2
CLIC2	XLOC_021755	2
CNKSR2	XLOC_022568	2
CSNK1B	XLOC_022429	2
DDX3Y	XLOC_022613	2
DOCK11	XLOC_021634	2
DRP2	XLOC_021810	2
DYNLT3	XLOC_022017	2
EIF1AX	XLOC_022050	2
EIF2S3Y	XLOC_022619	2
ELF4	XLOC_022128	2
ENOX2	XLOC_022133	2
F8	XLOC_021760	2
FAM127C	XLOC_022163	2
FAM155B	XLOC_022410	2
FAM199X	XLOC_021833	2
FAM58A	XLOC_022253	2
FANCB	XLOC_022074	2
FGD1	XLOC_022475	2
FLNA	XLOC_022268	2
FUNDC2	XLOC_022240	2
GATA1	XLOC_021936	2
GDI1	XLOC_021783	2
GNL3L	XLOC_021978	2
GPRASP1	XLOC_021796	2
GRIPAP1	XLOC_022441	2
HAUS7	XLOC_022252	2
IDS	XLOC_022197	2
IKBKG	XLOC_021786	2
IL13RA1	XLOC_022100	2
IL1RAPL1	XLOC_022535	2
IRAK1	XLOC_022264	2
KIAA1210	XLOC_022101	2
KLHL13	XLOC_022099	2
KLHL15	XLOC_022041	2
L1CAM	XLOC_022258	2
LOC100295854	XLOC_021989	2
LOC100298723	XLOC_021991	2
LOC100300937	XLOC_022563	2

Symbol	cuff_ID	Cluster #
LOC100847146	XLOC_022167	2
LOC100847396	XLOC_022152	2
LOC100847398	XLOC_022423	2
LOC100847475	XLOC_021694	2
LOC100847497	XLOC_022156	2
LOC100847577	XLOC_022460	2
LOC100847796	XLOC_022000	2
LOC100848511	XLOC_022134	2
LOC100848554	XLOC_022214	2
LOC100848761	XLOC_022502	2
LOC100848809	XLOC_022457	2
LOC537655	XLOC_022023	2
LOC541149	XLOC_021731	2
LOC615842	XLOC_022338	2
LOC618452	XLOC_021697	2
LOC781001	XLOC_021977	2
LOC782028	XLOC_022411	2
LOC785342	XLOC_022215	2
LOC786332	XLOC_022281	2
LOC786724	XLOC_021922	2
LOC787803	XLOC_021965	2
LONRF3	XLOC_021638	2
MAGED1	XLOC_022462	2
MAGED2	XLOC_021980	2
MAGEE2	XLOC_022378	2
MAGEH1	XLOC_022478	2
MAGT1	XLOC_021878	2
MAP7D2	XLOC_021719	2
MECP2	XLOC_022265	2
MID1	XLOC_022083	2
MORC4	XLOC_021836	2
MORF4L2	XLOC_022315	2
MSL3	XLOC_022605	2
MST4	XLOC_021678	2
MTM1	XLOC_021735	2
MXRA5	XLOC_022080	2
NAA10	XLOC_022260	2
NDUFA1	XLOC_021646	2
NRK	XLOC_021803	2
NXT2	XLOC_021844	2
OBP	XLOC_022628	2
PCYT1B	XLOC_022040	2
PGK1	XLOC_022373	2
PHKA1	XLOC_021893	2
PHKA2	XLOC_022059	2
PJA1	XLOC_021910	2
PLP2	XLOC_021943	2
PPEF1	XLOC_022570	2
PRAF2	XLOC_022443	2
PRDX4	XLOC_022561	2

Symbol	cuff_ID	Cluster #
PRICKLE3	XLOC_022447	2
RAB9A	XLOC_022586	2
RAP2C	XLOC_022139	2
RBBP7	XLOC_022063	2
RBM3	XLOC_021932	2
RBM41	XLOC_021838	2
RBMX2	XLOC_021672	2
RENBP	XLOC_022261	2
REPS2	XLOC_022574	2
RGN	XLOC_021917	2
RP2	XLOC_021914	2
RPL10	XLOC_021780	2
SAT1	XLOC_022560	2
SEPT6	XLOC_022105	2
SH3BGRL	XLOC_022355	2
SLC35A2	XLOC_022437	2
SRPX2	XLOC_022285	2
SUV39H1	XLOC_021935	2
SYTL4	XLOC_022290	2
SYTL5	XLOC_022503	2
TAF9B	XLOC_021877	2
TAZ	XLOC_021781	2
TBC1D8B	XLOC_022327	2
TCEAL8	XLOC_022313	2
TIMP1	XLOC_021924	2
TMEM187	XLOC_021776	2
TSPAN6	XLOC_021799	2
UPRT	XLOC_022379	2
USP9Y	XLOC_022606	2
USP9Y	XLOC_022607	2
UXT	XLOC_022426	2
WBP5	XLOC_021820	2
YIPF6	XLOC_022415	2
ZBED1	XLOC_022599	2
ZBTB33	XLOC_022115	2
ZC4H2	XLOC_021990	2
ZNF185	XLOC_021748	2
ZNF280C	XLOC_022130	2
ZNF449	XLOC_021696	2
	XLOC_021631	2
	XLOC_022181	2
ASMTL	XLOC_022097	3
BDA20	XLOC_022624	3
BMP15	XLOC_021962	3
COL4A6	XLOC_022332	3
CXHXorf36	XLOC_022003	3
CYLC1	XLOC_022354	3
CYSLTR1	XLOC_021874	3
EDA	XLOC_022409	3
FRMPD3	XLOC_022325	3

Symbol	cuff_ID	Cluster #
GLRA2	XLOC_022584	3
GUCY2F	XLOC_022335	3
IL13RA2	XLOC_022356	3
KLHL4	XLOC_021632	3
LOC100297941	XLOC_021660	3
LOC100336369	XLOC_021875	3
LOC100336599	XLOC_022595	3
LOC100336610	XLOC_022596	3
LOC100848108	XLOC_022465	3
LOC100848329	XLOC_022014	3
LOC100848501	XLOC_022434	3
LOC100848631	XLOC_022317	3
LOC519208	XLOC_021876	3
LOC526966	XLOC_022531	3
LOC615291	XLOC_021868	3
LOC615782	XLOC_021764	3
LOC616695	XLOC_022038	3
LOC617166	XLOC_021973	3
LOC786285	XLOC_021805	3
LOC786836	XLOC_022528	3
MAGEB3	XLOC_022539	3
NXF2	XLOC_022301	3
NXF3	XLOC_022311	3
OTUD6A	XLOC_022408	3
RAB9B	XLOC_022318	3
SLITRK4	XLOC_022190	3
TBX22	XLOC_022367	3
TCP11	XLOC_022304	3
TEX11	XLOC_021906	3
TLR8	XLOC_022601	3
XK	XLOC_022506	3
ZCCHC12	XLOC_021637	3
ZCCHC13	XLOC_022382	3
	XLOC_021650	3
	XLOC_022226	3
ACOT9	XLOC_022043	4
ACSL4	XLOC_022337	4
AFF2	XLOC_021724	4
APEX2	XLOC_021981	4
APOO	XLOC_022042	4
ARAF	XLOC_021923	4
ARHGEF9	XLOC_021995	4
ARSE	XLOC_022081	4
ASB11	XLOC_022072	4
ATP11C	XLOC_022182	4
ATP1B4	XLOC_022114	4
ATP6AP1	XLOC_021782	4
ATRX	XLOC_021879	4
BCAP31	XLOC_022255	4
BCOR	XLOC_022012	4

Symbol	cuff_ID	Cluster #
BRWD3	XLOC_021862	4
CASK	XLOC_022008	4
CCDC120	XLOC_021940	4
CCDC22	XLOC_021945	4
CDK16	XLOC_021920	4
CDKL5	XLOC_022571	4
CENPI	XLOC_021809	4
CLDN2	XLOC_022326	4
CSTF2	XLOC_021806	4
CTPS2	XLOC_022065	4
CUL4B	XLOC_021652	4
CXHXorf23	XLOC_021720	4
CXHXorf26	XLOC_022377	4
CXHXorf48	XLOC_022164	4
CXHXorf56	XLOC_022103	4
DDX3X	XLOC_022494	4
DIAPH2	XLOC_021792	4
DKC1	XLOC_022241	4
DLG3	XLOC_022401	4
DMD	XLOC_022024	4
DNASE1L1	XLOC_022269	4
EBP	XLOC_021930	4
EIF2S3	XLOC_022558	4
ELK1	XLOC_022425	4
EMD	XLOC_021779	4
ERAS	XLOC_021938	4
ERCC6L	XLOC_021897	4
FAM123B	XLOC_021994	4
FAM3A	XLOC_022273	4
FAM50A	XLOC_021784	4
FGF16	XLOC_022376	4
FMR1	XLOC_021722	4
FOXO4	XLOC_022400	4
FTSJ1	XLOC_021928	4
FUNDC1	XLOC_022004	4
G6PD	XLOC_022274	4
GEMIN8	XLOC_022075	4
GK	XLOC_022520	4
GLA	XLOC_022297	4
GPKOW	XLOC_022445	4
GPM6B	XLOC_022076	4
GYG2	XLOC_022597	4
HCCS	XLOC_022590	4
HCFC1	XLOC_022262	4
HDAC6	XLOC_021937	4
HDAC8	XLOC_021894	4
HMGN5	XLOC_021861	4
HNRNPH2	XLOC_021812	4
HPRT1	XLOC_021687	4
HSD17B10	XLOC_022467	4

Symbol	cuff_ID	Cluster #
HTATSF1	XLOC_021704	4
HUWE1	XLOC_022468	4
IDH3G	XLOC_022256	4
IGBP1	XLOC_022407	4
KAL1	XLOC_022091	4
KCND1	XLOC_022440	4
KDM5C	XLOC_022463	4
KDM6A	XLOC_022488	4
KIF4A	XLOC_022403	4
LAMP2	XLOC_021653	4
LAS1L	XLOC_021988	4
LOC100296441	XLOC_022192	4
LOC100300423	XLOC_022512	4
LOC100336547	XLOC_021934	4
LOC100336637	XLOC_021802	4
LOC100847211	XLOC_022037	4
LOC100847766	XLOC_022342	4
LOC100848000	XLOC_022369	4
LOC100848116	XLOC_022173	4
LOC100848605	XLOC_021825	4
LOC100848900	XLOC_021986	4
LOC100849028	XLOC_021890	4
LOC510362	XLOC_021998	4
LOC519242	XLOC_021744	4
LOC523454	XLOC_022611	4
LOC523963	XLOC_021662	4
LOC531038	XLOC_022394	4
LOC533597	XLOC_022579	4
LOC536163	XLOC_022472	4
LOC536163	XLOC_022473	4
LOC539973	XLOC_021974	4
LOC614207	XLOC_021880	4
LOC617695	XLOC_022198	4
LOC619027	XLOC_021692	4
LOC781022	XLOC_021845	4
LOC782177	XLOC_022280	4
LOC783300	XLOC_022380	4
LOC783344	XLOC_021793	4
LOC783577	XLOC_022048	4
LOC783730	XLOC_022162	4
LOC787088	XLOC_021702	4
LOC787822	XLOC_022199	4
MAGEB16	XLOC_022515	4
MAMLD1	XLOC_021734	4
MAP7D3	XLOC_022171	4
MBTPS2	XLOC_022567	4
MCTS1	XLOC_022113	4
MED12	XLOC_022399	4
MED14	XLOC_022010	4
MMGT1	XLOC_022170	4

Symbol	cuff_ID	Cluster #
MOSPD1	XLOC_022160	4
MOSPD2	XLOC_022583	4
MPP1	XLOC_021761	4
MSN	XLOC_022484	4
MTCP1	XLOC_021759	4
MTCP1NB	XLOC_021758	4
MTMR1	XLOC_021736	4
NDUFB11	XLOC_022419	4
NLGN3	XLOC_022398	4
NONO	XLOC_022396	4
NSDHL	XLOC_021747	4
OCRL	XLOC_021663	4
OFD1	XLOC_022585	4
OGT	XLOC_022391	4
OTUD5	XLOC_022439	4
PAK3	XLOC_021848	4
PDHA1	XLOC_022569	4
PDK3	XLOC_022555	4
PDZD11	XLOC_021907	4
PGRMC1	XLOC_021640	4
PHF16	XLOC_021916	4
PIM2	XLOC_022438	4
POLA1	XLOC_022554	4
PPP1R3F	XLOC_021946	4
PQBP1	XLOC_021939	4
PRPS1	XLOC_022324	4
PRPS2	XLOC_022085	4
PSMD10	XLOC_022330	4
RBM10	XLOC_021918	4
RBMX	XLOC_022174	4
RBMX2	XLOC_021855	4
RLIM	XLOC_021885	4
RNF113A	XLOC_022108	4
RPGR	XLOC_022015	4
RPL36A	XLOC_021811	4
RPL39	XLOC_022106	4
RPS4X	XLOC_021896	4
RPS6KA3	XLOC_022049	4
RPS6KA6	XLOC_021860	4
SASH3	XLOC_021665	4
SH3KBP1	XLOC_022051	4
SLC10A3	XLOC_022272	4
SLC25A43	XLOC_021641	4
SLC25A5	XLOC_021642	4
SLC7A3	XLOC_021905	4
SLC9A6	XLOC_021700	4
SMC1A	XLOC_022466	4
SMS	XLOC_022566	4
SNX12	XLOC_021904	4
SSR4	XLOC_021774	4

Symbol	cuff_ID	Cluster #
STAG2	XLOC_021657	4
STS	XLOC_022622	4
SYAP1	XLOC_022576	4
TAF1	XLOC_022392	4
TBC1D25	XLOC_021931	4
TBL1X	XLOC_022620	4
TCEAL4	XLOC_021823	4
TFE3	XLOC_022442	4
THOC2	XLOC_022116	4
TIMM17B	XLOC_022436	4
TIMM8A	XLOC_022294	4
TMEM27	XLOC_022066	4
TMEM35	XLOC_021808	4
TMLHE	XLOC_021754	4
TMSB4X	XLOC_022600	4
TRAPPC2	XLOC_022077	4
TSPAN7	XLOC_022499	4
TSPYL2	XLOC_021972	4
TSR2	XLOC_021976	4
TXLNG	XLOC_022575	4
UBA1	XLOC_021919	4
UBE2A	XLOC_021643	4
UBL4A	XLOC_022271	4
UBQLN2	XLOC_021985	4
UPF3B	XLOC_022107	4
USP11	XLOC_021921	4
USP27X	XLOC_021949	4
USP9X	XLOC_022495	4
UTP14A	XLOC_021667	4
UTY	XLOC_022612	4
VAMP7	XLOC_022234	4
VBP1	XLOC_022237	4
VMA21	XLOC_021739	4
WDR44	XLOC_021633	4
WDR45	XLOC_022444	4
WNK3	XLOC_022474	4
XIAP	XLOC_021656	4
ZDHHC9	XLOC_022127	4
ZFX	XLOC_022557	4
ZMYM3	XLOC_021901	4
ZNF75D	XLOC_021695	4
ZNF75D	XLOC_022165	4
ZNF81	XLOC_022422	4
ZRSR2	XLOC_022580	4
ZRSR2Y	XLOC_022609	4
	XLOC_021737	4
	XLOC_022471	4
ACE2	XLOC_022068	5
ARHGAP36	XLOC_021674	5
BEX2	XLOC_022284	5

Symbol	cuff_ID	Cluster #
BTK	XLOC_022295	5
C9HXorf21	XLOC_022026	5
CAPN6	XLOC_022347	5
CITED1	XLOC_021895	5
CT47B1	XLOC_021649	5
CXHXorf22	XLOC_022513	5
CXHXorf41	XLOC_022328	5
CYBB	XLOC_022505	5
DACH2	XLOC_022361	5
DGAT2L6	XLOC_022406	5
FAM46D	XLOC_022366	5
FGF13	XLOC_022177	5
FMR1NB	XLOC_021723	5
FOXP3	XLOC_022450	5
GABRA3	XLOC_022209	5
GABRQ	XLOC_021745	5
GDPD2	XLOC_022402	5
GJB1	XLOC_022397	5
GPR119	XLOC_022131	5
GPR143	XLOC_022089	5
GPR34	XLOC_022493	5
GPR50	XLOC_021738	5
GPR64	XLOC_022058	5
IL2RG	XLOC_021902	5
KLF8	XLOC_021984	5
LDOC1	XLOC_021712	5
LOC100126054	XLOC_022480	5
LOC100295281	XLOC_021666	5
LOC100296348	XLOC_021966	5
LOC100296541	XLOC_022454	5
LOC100297099	XLOC_022459	5
LOC100298021	XLOC_022201	5
LOC100298655	XLOC_021717	5
LOC100300091	XLOC_022625	5
LOC100300693	XLOC_021682	5
LOC100336532	XLOC_021636	5
LOC100337093	XLOC_022168	5
LOC100337225	XLOC_022527	5
LOC100847166	XLOC_021659	5
LOC100847397	XLOC_022228	5
LOC100847564	XLOC_021898	5
LOC100847679	XLOC_022519	5
LOC100847953	XLOC_022322	5
LOC520085	XLOC_022537	5
LOC520682	XLOC_022496	5
LOC523644	XLOC_022617	5
LOC529992	XLOC_022238	5
LOC538872	XLOC_022489	5
LOC613515	XLOC_022298	5
LOC614768	XLOC_021870	5

Symbol	cuff_ID	Cluster #
LOC615983	XLOC_022232	5
LOC616431	XLOC_021830	5
LOC616821	XLOC_021839	5
LOC617499	XLOC_022180	5
LOC617503	XLOC_021639	5
LOC618023	XLOC_022276	5
LOC618701	XLOC_022029	5
LOC781486	XLOC_022529	5
LOC781876	XLOC_021714	5
LOC783362	XLOC_021681	5
LOC783713	XLOC_022087	5
LOC783993	XLOC_021684	5
LOC784285	XLOC_022196	5
LOC784572	XLOC_022556	5
LOC785658	XLOC_022431	5
LOC786942	XLOC_021683	5
LOC787130	XLOC_021964	5
LOC787476	XLOC_022132	5
LOC788399	XLOC_022521	5
LOC789808	XLOC_021645	5
MAGEA9	XLOC_021746	5
MAOA	XLOC_022491	5
MAOB	XLOC_022007	5
MAP3K15	XLOC_022052	5
NGFRAP1	XLOC_021821	5
NOX1	XLOC_022291	5
NUDT10	XLOC_021963	5
NUDT11	XLOC_022456	5
NXF3	XLOC_021798	5
PABPC1L2A	XLOC_021892	5
PAGE4	XLOC_021948	5
PDZD4	XLOC_022257	5
RAB39B	XLOC_021757	5
SAGE1	XLOC_021699	5
SFRS17A	XLOC_022102	5
SLC16A2	XLOC_022381	5
SLITRK2	XLOC_022193	5
SPRY3	XLOC_022235	5
SYN1	XLOC_021925	5
TGIF2LX	XLOC_022279	5
TKTL1	XLOC_021778	5
TLR7	XLOC_022602	5
TRO	XLOC_021979	5
TRPC5	XLOC_022349	5
VGLL1	XLOC_021705	5
XKRX	XLOC_022292	5
ZC3H12B	XLOC_022485	5
ZIC3	XLOC_021708	5
	XLOC_021947	5
ASB9	XLOC_022073	6

Symbol	cuff_ID	Cluster #
BMX	XLOC_022582	6
CXHXorf30	XLOC_022511	6
EFHC2	XLOC_022005	6
EIF1AY	XLOC_022618	6
GLRA4	XLOC_022316	6
GRPR	XLOC_022578	6
KCNE1L	XLOC_022336	6
LOC100126053	XLOC_022278	6
LOC100296832	XLOC_022490	6
LOC100300042	XLOC_022509	6
LOC100300684	XLOC_022112	6
LOC100301024	XLOC_022283	6
LOC100336547	XLOC_022433	6
LOC100336830	XLOC_022233	6
LOC100847174	XLOC_022221	6
LOC100847435	XLOC_022064	6
LOC100847519	XLOC_021689	6
LOC100847614	XLOC_022461	6
LOC100847678	XLOC_021967	6
LOC100847680	XLOC_022616	6
LOC100848901	XLOC_022056	6
LOC514871	XLOC_022248	6
LOC520057	XLOC_022492	6
LOC523458	XLOC_021831	6
LOC532367	XLOC_022514	6
LOC614781	XLOC_022245	6
LOC616656	XLOC_022119	6
LOC617475	XLOC_021756	6
LOC617999	XLOC_021763	6
LOC781489	XLOC_022548	6
LOC781841	XLOC_022189	6
LOC782892	XLOC_022110	6
LOC783491	XLOC_021680	6
LOC785684	XLOC_022111	6
LOC786510	XLOC_021832	6
LOC786698	XLOC_022319	6
MAGEB16	XLOC_022020	6
MAGEB18	XLOC_022553	6
MGC133764	XLOC_022523	6
MGC148328	XLOC_022194	6
PHEX	XLOC_022565	6
PRDM7	XLOC_022277	6
PRDM9	XLOC_022614	6
PRRG1	XLOC_022333	6
PRRG3	XLOC_021741	6
RAI2	XLOC_022062	6
SH2D1A	XLOC_021658	6
SH3KBP1	XLOC_021721	6
SOWAHD	XLOC_021644	6
SPIN2B	XLOC_022481	6

Symbol	cuff_ID	Cluster #
SYP	XLOC_022448	6
TCEAL7	XLOC_021819	6
TEX28	XLOC_022267	6
USP26	XLOC_022143	6
ARHGAP6	XLOC_022078	7
ARMCX1	XLOC_021814	7
ARMCX6	XLOC_022299	7
BGN	XLOC_021767	7
CCDC160	XLOC_021685	7
CD40LG	XLOC_021706	7
CXCR3	XLOC_021900	7
DCX	XLOC_022348	7
FAM70A	XLOC_021654	7
FRMD7	XLOC_022138	7
GABRE	XLOC_022204	7
GPC3	XLOC_022145	7
GRIA3	XLOC_021655	7
LOC100138824	XLOC_022140	7
LOC100295484	XLOC_022359	7
LOC100299061	XLOC_022341	7
LOC100336905	XLOC_022339	7
LOC100337493	XLOC_021858	7
LOC100847900	XLOC_021669	7
LOC100848206	XLOC_022345	7
LOC100848225	XLOC_022346	7
LOC531026	XLOC_021713	7
LOC784453	XLOC_022123	7
LPAR4	XLOC_022371	7
MAGED4B	XLOC_021970	7
MIR374B	XLOC_021888	7
PABPC5	XLOC_021787	7
PCDH11Y	XLOC_021789	7
PLXNB3	XLOC_021772	7
RIBC1	XLOC_021975	7
SPACA5	XLOC_022451	7
SRPX	XLOC_022016	7
TMEM47	XLOC_022021	7
TSC22D3	XLOC_021835	7
ZXDB	XLOC_022486	7
	XLOC_021701	7
ACTRT1	XLOC_022124	8
APLN	XLOC_022126	8
ARHGAP6	XLOC_022086	8
ARX	XLOC_022039	8
AVPR2	XLOC_021775	8
CHST7	XLOC_021913	8
CNGA2	XLOC_021743	8
CXHXorf64	XLOC_021661	8
ERI1	XLOC_021797	8
FRMPD4	XLOC_022604	8

Symbol	cuff_ID	Cluster #
GPR101	XLOC_022176	8
GPR174	XLOC_022368	8
IGSF1	XLOC_022136	8
IRS4	XLOC_022334	8
KIR2DL5A	XLOC_021733	8
KIR3DL2	XLOC_022244	8
KLHL34	XLOC_022047	8
LOC100139604	XLOC_022166	8
LOC100140878	XLOC_021915	8
LOC100296529	XLOC_021715	8
LOC100335456	XLOC_021865	8
LOC100335650	XLOC_022243	8
LOC100336670	XLOC_021944	8
LOC100336731	XLOC_021753	8
LOC100337198	XLOC_022615	8
LOC100337353	XLOC_022532	8
LOC100847173	XLOC_021716	8
LOC100847299	XLOC_022225	8
LOC100847442	XLOC_022230	8
LOC100848714	XLOC_021828	8
LOC505052	XLOC_022627	8
LOC507302	XLOC_022036	8
LOC515006	XLOC_021711	8
LOC519648	XLOC_022385	8
LOC526223	XLOC_021850	8
LOC528106	XLOC_021676	8
LOC532022	XLOC_022517	8
LOC615101	XLOC_021791	8
LOC618364	XLOC_022547	8
LOC619156	XLOC_022009	8
LOC782178	XLOC_022191	8
LOC784097	XLOC_022383	8
LOC788278	XLOC_022533	8
MAGEA10	XLOC_022208	8
MAGEA11	XLOC_021709	8
MAGEB4	XLOC_022524	8
MCART6	XLOC_022320	8
MGC140080	XLOC_022386	8
MGC151921	XLOC_022623	8
MGC152340	XLOC_021899	8
MIR3431	XLOC_022205	8
PABPC1L2A	XLOC_022387	8
PFKFB1	XLOC_022476	8
PLAC1	XLOC_022158	8
PLP1	XLOC_021827	8
RS1	XLOC_022060	8
SERPINA7	XLOC_022288	8
SLC6A14	XLOC_022098	8
TNMD	XLOC_022286	8
	XLOC_022120	8

Symbol	cuff_ID	Cluster #
ALAS2	XLOC_022477	9
ARL13A	XLOC_021807	9
AWAT2	XLOC_021909	9
CACNA1F	XLOC_022449	9
COL4A5	XLOC_021842	9
COX7B	XLOC_022375	9
DGKK	XLOC_022453	9
GAB3	XLOC_021762	9
HDHD1	XLOC_022093	9
LOC100297779	XLOC_021968	9
LOC100299005	XLOC_021969	9
LOC100301478	XLOC_021961	9
LOC100337148	XLOC_022305	9
LOC100847220	XLOC_022358	9
LOC100847335	XLOC_021864	9
LOC100848079	XLOC_022236	9
LOC100848737	XLOC_022501	9
LOC516074	XLOC_021837	9
LOC617648	XLOC_021749	9
LOC781152	XLOC_021846	9
LOC783509	XLOC_022289	9
LOC786258	XLOC_021822	9
MAGIX	XLOC_021942	9
MBNL3	XLOC_022141	9
NKRF	XLOC_022104	9
NRK	XLOC_022287	9
NXF3	XLOC_022306	9
PCDH19	XLOC_021800	9
PIGA	XLOC_022071	9
PIN4	XLOC_022388	9
PNCK	XLOC_022254	9
PNPLA4	XLOC_022092	9
PORCN	XLOC_021929	9
PRPS2	XLOC_022603	9
RNF128	XLOC_022308	9
SLC6A8	XLOC_021770	9
SLC9A7	XLOC_022418	9
STARD8	XLOC_022414	9
SYN1	XLOC_022421	9
TAB3	XLOC_022025	9
TM9SF2	XLOC_021707	9
TMEM164	XLOC_021847	9
XIST	XLOC_021891	9
XPNPEP2	XLOC_021664	9
ZNF674	XLOC_022487	9
	XLOC_021960	9
