# Supplementary material for: Characterization of X-Chromosome Gene Expression in Bovine Blastocysts Derived by In vitro Fertilization and Somatic Cell Nuclear Transfer
Source: Front Genet. 2017 Apr 10;8:42. doi: 10.3389/fgene.2017.00042 (PMC5385346; doi:10.3389/fgene.2017.00042)
Supplement: Supplementary Table S2 — xDEGs between male vs. female blastocysts. [file Table2.PDF]

Table S2. xDEGs between IVF male and female blastocysts

Symbol	mean FPKM of male IVF	mean FPKM of female IVF	log_2(female/male)	*p* -val	*q* -val
ACRC	0.28	2.46	3.16	3.45E-02	1.48E-01
AFF2	0.43	1.79	2.07	4.50E-03	3.58E-02
AIFM1	17.34	30.46	0.81	9.00E-03	5.87E-02
AMOT	71.27	133.35	0.90	3.00E-04	4.56E-03
APEX2	10.88	23.29	1.10	3.65E-03	3.08E-02
APLN	0.00	5.32	Inf	4.60E-03	3.64E-02
ATG4A	7.60	18.41	1.28	1.53E-02	8.46E-02
ATP11C	21.55	58.67	1.44	5.00E-05	1.05E-03
ATP6AP1	52.84	112.03	1.08	5.00E-05	1.05E-03
ATP6AP2	14.93	38.95	1.38	1.50E-04	2.62E-03
ATRX	32.45	65.24	1.01	5.00E-05	1.05E-03
BCOR	17.75	27.05	0.61	2.23E-02	1.10E-01
BCORL1	4.43	8.71	0.98	5.50E-03	4.13E-02
BEX2	0.59	7.00	3.58	2.36E-02	1.14E-01
BRCC3	4.22	8.98	1.09	3.60E-02	1.52E-01
BRWD3	5.28	13.97	1.40	5.00E-05	1.05E-03
C1GALT1C1	4.13	9.38	1.18	2.01E-02	1.02E-01
CAPN6	3.58	12.02	1.75	2.45E-03	2.30E-02
CASK	108.23	194.67	0.85	3.05E-03	2.70E-02
CCDC120	5.16	10.09	0.97	9.95E-03	6.30E-02
CD99	60.86	37.61	-0.69	1.06E-02	6.61E-02
CDK16	31.00	57.62	0.89	1.65E-03	1.71E-02
CDKL5	12.55	34.93	1.48	5.00E-05	1.05E-03
CENPI	10.28	17.88	0.80	7.45E-03	5.13E-02
CITED1	11.42	57.53	2.33	5.00E-05	1.05E-03
CSTF2	35.74	112.65	1.66	5.00E-05	1.05E-03
CT47B1	7.36	47.22	2.68	5.00E-05	1.05E-03
CUL4B	8.89	16.94	0.93	1.55E-03	1.63E-02
CXHXorf23	16.56	42.81	1.37	5.00E-05	1.05E-03
CXHXorf57	1.76	11.66	2.73	5.00E-05	1.05E-03
DDX3X	88.24	127.54	0.53	2.57E-02	1.21E-01
DNASE1L1	18.98	31.92	0.75	1.78E-02	9.39E-02
EIF1AX	19.34	38.71	1.00	2.27E-02	1.11E-01
EIF2S3Y	4.60	0.00	-Inf	5.00E-05	1.05E-03
ELF4	21.20	61.69	1.54	5.00E-05	1.05E-03
ELK1	12.97	25.75	0.99	5.15E-03	3.95E-02
EMD	17.98	49.72	1.47	2.00E-04	3.30E-03
FAM50A	90.48	141.48	0.64	1.46E-02	8.18E-02
FAM58A	5.08	12.98	1.35	1.85E-02	9.67E-02
FLNA	16.32	28.55	0.81	1.95E-03	1.94E-02
FMR1	7.42	18.33	1.30	5.00E-05	1.05E-03
FOXO4	1.16	3.37	1.54	2.44E-02	1.17E-01
FTSJ1	24.25	41.41	0.77	5.50E-03	4.13E-02
FUNDC1	21.53	41.68	0.95	1.42E-02	8.03E-02
FUNDC2	35.07	72.70	1.05	4.25E-03	3.44E-02
G6PD	36.15	71.03	0.97	3.00E-04	4.56E-03
GABRA3	25.70	106.37	2.05	5.00E-05	1.05E-03
GDI1	23.28	44.29	0.93	1.20E-03	1.34E-02
GDPD2	62.70	33.55	-0.90	1.80E-03	1.82E-02
GK	9.46	23.74	1.33	9.00E-04	1.08E-02
GLA	3.80	10.73	1.50	4.35E-03	3.50E-02
GNL3L	12.93	38.91	1.59	5.00E-05	1.05E-03

GPKOW	10.20	28.97	1.51	5.00E-05	1.05E-03
GPR64	4.10	13.63	1.73	5.00E-05	1.05E-03
GRIPAP1	8.74	29.54	1.76	5.00E-05	1.05E-03
GYG2	22.97	15.27	-0.59	4.49E-02	1.76E-01
HCFC1	51.61	91.33	0.82	1.00E-03	1.17E-02
HDAC6	82.37	121.54	0.56	2.69E-02	1.25E-01
HDHD1	13.91	24.32	0.81	2.08E-02	1.05E-01
HNRNPH2	15.80	37.66	1.25	2.00E-04	3.30E-03
HTATSF1	14.70	47.00	1.68	5.00E-05	1.05E-03
IDH3G	96.42	176.11	0.87	8.00E-04	9.81E-03
IL13RA1	5.73	13.39	1.22	1.48E-02	8.30E-02
KDM6A	35.51	71.10	1.00	5.00E-05	1.05E-03
KIF4A	35.30	52.31	0.57	3.04E-02	1.35E-01
KLHL13	1.84	5.50	1.58	2.99E-02	1.34E-01
KLHL15	13.19	49.98	1.92	5.00E-05	1.05E-03
L1CAM	3.11	7.77	1.32	3.40E-03	2.93E-02
LAMP2	112.94	179.70	0.67	1.25E-02	7.37E-02
LDOC1	15.21	56.31	1.89	5.00E-05	1.05E-03
LOC100297099	11.41	30.94	1.44	3.24E-02	1.42E-01
LOC100299005	0.69	10.49	3.93	4.41E-02	1.73E-01
LOC100300684	0.32	4.47	3.82	3.64E-02	1.53E-01
LOC100336731	0.00	2.45	Inf	9.25E-03	5.99E-02
LOC100847146	10.23	22.97	1.17	5.65E-03	4.21E-02
LOC100847299	0.00	29.24	Inf	5.00E-05	1.05E-03
LOC100847564	1.40	4.24	1.60	3.53E-02	1.50E-01
LOC100848605	196.75	27.82	-2.82	5.00E-05	1.05E-03
LOC100848900	4.67	15.81	1.76	1.37E-02	7.87E-02
LOC520085	10.38	29.09	1.49	3.35E-03	2.90E-02
LOC523454	98.21	229.85	1.23	5.00E-05	1.05E-03
LOC613515	4.72	11.87	1.33	4.79E-02	1.83E-01
LOC615842	4.00	12.21	1.61	2.13E-02	1.06E-01
LOC616431	0.72	6.76	3.23	2.74E-02	1.26E-01
LOC616695	0.07	5.87	6.32	2.07E-02	1.05E-01
LOC618023	232.62	456.99	0.97	4.21E-02	1.68E-01
LOC783344	2.19	10.35	2.24	4.45E-03	3.56E-02
LOC783509	170.00	2226.45	3.71	5.00E-05	1.05E-03
LOC783577	241.07	550.26	1.19	5.00E-05	1.05E-03
LOC786836	0.41	2.75	2.74	4.85E-02	1.85E-01
LOC787476	2.05	14.27	2.80	5.00E-05	1.05E-03
MAGEB16	0.50	3.59	2.85	3.93E-02	1.61E-01
MAGED1	59.23	105.89	0.84	8.00E-04	9.81E-03
MAP7D3	42.20	72.02	0.77	3.95E-03	3.26E-02
MBNL3	33.60	81.75	1.28	1.00E-04	1.88E-03
MCTS1	3.77	17.27	2.19	3.00E-03	2.67E-02
MECP2	2.20	4.44	1.01	2.80E-03	2.53E-02
MED14	17.37	36.22	1.06	1.00E-04	1.88E-03
MID1	4.49	8.93	0.99	9.85E-03	6.26E-02
MORC4	0.74	4.82	2.70	8.50E-04	1.03E-02
MORF4L2	12.09	30.35	1.33	5.50E-04	7.35E-03
MOSPD1	5.72	15.17	1.41	1.00E-04	1.88E-03
MPP1	21.62	47.70	1.14	5.00E-05	1.05E-03
MSL3	12.48	34.50	1.47	1.50E-04	2.62E-03
MSN	423.34	958.13	1.18	5.00E-05	1.05E-03
MTM1	0.96	3.35	1.80	8.70E-03	5.73E-02
NONO	240.35	499.06	1.05	5.00E-05	1.05E-03
NSDHL	11.19	33.18	1.57	1.00E-04	1.88E-03
NXT2	3.03	10.17	1.75	1.50E-03	1.59E-02
OCRL	43.73	91.69	1.07	5.00E-05	1.05E-03
OTUD5	44.86	90.75	1.02	3.00E-04	4.56E-03

PDK3	28.39	49.15	0.79	4.55E-03	3.61E-02
PGK1	24.94	40.46	0.70	3.20E-02	1.41E-01
PGRMC1	48.30	83.14	0.78	6.05E-03	4.42E-02
PHF16	4.14	9.90	1.26	4.10E-03	3.35E-02
PHF8	49.18	93.09	0.92	3.00E-04	4.56E-03
PHKA1	1.08	4.80	2.15	5.65E-03	4.21E-02
PHKA2	3.23	12.99	2.01	5.00E-05	1.05E-03
PLP2	7.43	22.60	1.60	1.30E-03	1.43E-02
POLA1	10.63	26.23	1.30	5.00E-05	1.05E-03
PORCN	2.43	11.17	2.20	4.75E-03	3.72E-02
PQBP1	24.03	39.28	0.71	4.02E-02	1.63E-01
PRICKLE3	5.46	11.99	1.13	2.70E-02	1.25E-01
PRPS1	8.22	23.30	1.50	5.00E-05	1.05E-03
PRPS2	1.72	8.69	2.33	1.45E-03	1.55E-02
PRPS2	17.82	56.66	1.67	2.25E-02	1.11E-01
PSMD10	32.18	80.77	1.33	1.50E-04	2.62E-03
RAP2C	0.88	2.37	1.42	3.29E-02	1.43E-01
RBBP7	49.82	130.53	1.39	5.00E-05	1.05E-03
RBMX	34.75	57.56	0.73	1.42E-02	8.05E-02
RBMX2	7.86	25.14	1.68	3.00E-04	4.56E-03
RBMX2	16.81	29.50	0.81	3.51E-02	1.49E-01
RENBP	2.49	8.22	1.73	9.10E-03	5.92E-02
RPL39	50.23	119.69	1.25	3.55E-03	3.02E-02
RPS4X	1460.64	2214.53	0.60	1.98E-02	1.01E-01
RPS6KA3	14.53	24.26	0.74	2.40E-02	1.16E-01
SAT1	3.65	20.99	2.52	6.00E-04	7.86E-03
SEPT6	10.52	19.95	0.92	1.11E-02	6.82E-02
SH3KBP1	25.57	39.36	0.62	3.59E-02	1.51E-01
SLC25A43	28.25	45.71	0.69	3.20E-02	1.41E-01
SLC25A5	203.29	519.10	1.35	5.00E-05	1.05E-03
SLC35A2	4.20	11.27	1.42	3.40E-03	2.93E-02
SLC6A8	127.38	273.35	1.10	3.50E-04	5.15E-03
SLC9A6	4.28	10.74	1.33	6.00E-04	7.86E-03
SLITRK2	12.15	19.95	0.72	2.28E-02	1.12E-01
SMC1A	104.71	286.70	1.45	5.00E-05	1.05E-03
SNX12	15.64	29.17	0.90	4.60E-03	3.64E-02
SRPX2	4.34	16.50	1.93	5.50E-04	7.35E-03
SSR4	129.04	215.00	0.74	1.49E-02	8.32E-02
TAF1	9.24	20.03	1.12	5.00E-05	1.05E-03
TAF9B	2.31	7.16	1.63	1.57E-02	8.60E-02
TBC1D25	4.81	11.16	1.22	5.65E-03	4.21E-02
TFE3	33.64	67.93	1.01	2.50E-04	3.94E-03
THOC2	25.41	39.01	0.62	1.92E-02	9.92E-02
TIMP1	23.66	110.53	2.22	5.00E-05	1.05E-03
TM9SF2	3.72	0.80	-2.21	1.05E-03	1.21E-02
TMSB4X	25.54	52.00	1.03	2.04E-02	1.03E-01
TSPAN7	4.11	10.92	1.41	9.80E-03	6.24E-02
TSR2	24.75	61.61	1.32	2.50E-04	3.94E-03
TXLNG	11.11	26.78	1.27	2.50E-04	3.94E-03
UBA1	198.78	301.88	0.60	1.18E-02	7.10E-02
UBQLN2	5.27	22.85	2.12	5.00E-05	1.05E-03
UPRT	4.32	64.00	3.89	5.00E-05	1.05E-03
USP11	30.62	47.21	0.62	1.56E-02	8.59E-02
USP9X	13.97	31.12	1.16	5.00E-05	1.05E-03
USP9Y	1.88	0.00	-Inf	5.00E-05	1.05E-03
USP9Y	2.95	0.00	-Inf	1.26E-02	7.44E-02
UTP14A	25.22	50.90	1.01	5.00E-04	6.82E-03
VMA21	10.05	18.00	0.84	4.15E-03	3.38E-02
WBP5	0.38	6.99	4.21	1.70E-02	9.09E-02

WDR44	33.15	52.58	0.67	1.14E-02	6.93E-02
WDR45	53.67	86.42	0.69	1.42E-02	8.03E-02
WNK3	2.27	5.25	1.21	5.30E-03	4.02E-02
XIAP	42.83	19.65	-1.12	1.25E-03	1.38E-02
XIST	6.49	60.34	3.22	5.00E-05	1.05E-03
ZBED1	7.42	3.60	-1.04	4.70E-03	3.69E-02
ZBTB33	2.99	5.74	0.94	1.09E-02	6.70E-02
ZC4H2	3.64	7.17	0.98	4.65E-02	1.80E-01
ZDHHC9	44.71	113.14	1.34	5.00E-05	1.05E-03
ZFX	31.44	46.67	0.57	2.44E-02	1.17E-01
ZNF182	0.48	1.38	1.52	1.24E-02	7.35E-02
ZRSR2Y	4.07	0.00	-Inf	5.00E-05	1.05E-03

xDEGs between SCNT male and female blastocysts

Symbol	mean FPKM of male SCNT	mean FPKM of female SCNT	$\log_2$(female/male)	*p*-val	*q*-val
ACOT9	21.44	11.71	-0.87	1.88E-02	9.76E-02
ACSL4	97.39	37.16	-1.39	1.00E-04	1.88E-03
ALAS2	4.21	1.52	-1.47	2.61E-02	1.22E-01
AMOT	57.62	202.34	1.81	5.00E-05	1.05E-03
APEX2	9.47	20.49	1.11	4.65E-03	3.66E-02
APOO	16.23	5.92	-1.45	3.29E-02	1.43E-01
ARAF	24.34	78.53	1.69	5.00E-05	1.05E-03
ARSE	16.51	6.69	-1.30	3.60E-03	3.05E-02
ATP11C	23.11	46.07	1.00	3.50E-04	5.15E-03
ATP6AP1	41.74	105.99	1.34	5.00E-05	1.05E-03
ATP6AP2	39.73	25.62	-0.63	3.99E-02	1.62E-01
BCOR	16.07	35.00	1.12	5.00E-05	1.05E-03
BRWD3	7.91	14.21	0.85	5.20E-03	3.97E-02
C1GALT1C1	3.37	7.10	1.08	3.51E-02	1.49E-01
CAPN6	3.25	6.93	1.09	2.69E-02	1.25E-01
CCDC22	6.43	20.61	1.68	2.50E-04	3.94E-03
CDK16	26.39	70.66	1.42	5.00E-05	1.05E-03
CDKL5	6.03	20.54	1.77	5.00E-05	1.05E-03
CENPI	10.81	16.89	0.64	3.25E-02	1.42E-01
CHM	3.98	6.74	0.76	2.89E-02	1.31E-01
CITED1	16.97	32.81	0.95	3.05E-02	1.36E-01
CT47B1	4.04	18.67	2.21	5.00E-05	1.05E-03
CTPS2	1.96	8.26	2.07	6.50E-03	4.65E-02
CUL4B	7.39	14.46	0.97	2.20E-03	2.12E-02
CXHXorf26	17.83	9.10	-0.97	2.47E-02	1.18E-01
DLG3	10.25	5.47	-0.90	7.10E-03	4.96E-02
EIF2S3	43.05	26.52	-0.70	2.92E-02	1.32E-01
EIF2S3Y	6.24	0.00	-Inf	5.00E-05	1.05E-03
ELF4	18.72	88.17	2.24	5.00E-05	1.05E-03
ELK1	10.51	21.60	1.04	2.70E-03	2.47E-02
EMD	25.99	61.90	1.25	3.50E-04	5.15E-03
FAM123B	12.88	31.54	1.29	5.00E-05	1.05E-03
FAM3A	4.29	16.04	1.90	1.50E-04	2.62E-03
FAM58A	5.48	13.33	1.28	1.17E-02	7.05E-02
FLNA	8.95	22.06	1.30	5.00E-05	1.05E-03

FMR1	10.27	16.96	0.72	2.46E-02	1.17E-01
FMR1NB	0.28	2.76	3.29	2.25E-02	1.10E-01
FTSJ1	31.69	51.02	0.69	1.33E-02	7.72E-02
FUNDC2	21.61	42.28	0.97	3.74E-02	1.56E-01
G6PD	25.99	72.00	1.47	5.00E-05	1.05E-03
GABRA3	26.91	114.84	2.09	5.00E-05	1.05E-03
GDI1	16.50	34.61	1.07	6.00E-04	7.86E-03
GLA	4.68	12.26	1.39	1.02E-02	6.39E-02
GNL3L	11.44	27.84	1.28	1.00E-04	1.88E-03
GPKOW	8.96	22.81	1.35	5.00E-05	1.05E-03
GPR64	5.21	9.96	0.93	2.78E-02	1.27E-01
GRIPAP1	9.42	16.82	0.84	1.70E-02	9.13E-02
HAUS7	2.84	7.33	1.37	3.95E-02	1.62E-01
HCCS	104.01	59.21	-0.81	9.10E-03	5.92E-02
HCFC1	31.40	103.92	1.73	5.00E-05	1.05E-03
HDAC6	68.91	128.61	0.90	7.50E-04	9.35E-03
HDHD1	8.17	16.08	0.98	8.90E-03	5.83E-02
HUWE1	191.40	397.98	1.06	9.50E-04	1.12E-02
IDH3G	43.44	190.47	2.13	5.00E-05	1.05E-03
IGBP1	24.44	13.99	-0.81	1.70E-02	9.13E-02
IL13RA1	5.59	15.87	1.51	3.40E-03	2.93E-02
IRAK1	1.31	3.17	1.28	4.39E-02	1.73E-01
KCND1	0.91	2.54	1.48	1.96E-02	1.01E-01
KDM5C	26.77	48.31	0.85	1.05E-03	1.21E-02
KDM6A	24.30	81.11	1.74	5.00E-05	1.05E-03
KIF4A	34.19	62.56	0.87	1.05E-03	1.21E-02
KLHL15	12.74	46.56	1.87	5.00E-05	1.05E-03
LDOC1	13.70	38.07	1.47	2.00E-04	3.30E-03
LOC100847211	3.46	20.56	2.57	6.00E-04	7.86E-03
LOC100847299	0.82	14.51	4.15	1.87E-02	9.73E-02
LOC100847435	0.00	1.52	Inf	1.76E-02	9.31E-02
LOC100847766	14.02	0.00	-Inf	2.14E-02	1.07E-01
LOC100847953	11.82	40.65	1.78	3.50E-04	5.15E-03
LOC100848079	7.50	3.76	-1.00	2.14E-02	1.07E-01
LOC100848605	469.17	73.50	-2.67	5.00E-05	1.05E-03
LOC523454	89.17	133.73	0.58	1.87E-02	9.73E-02
LOC523963	12.20	65.66	2.43	5.00E-05	1.05E-03
LOC536163	18.31	6.38	-1.52	2.40E-03	2.26E-02
LOC614207	40.70	21.81	-0.90	1.55E-03	1.63E-02
LOC783362	0.81	14.53	4.17	1.06E-02	6.58E-02
LOC783577	312.52	536.50	0.78	1.70E-03	1.75E-02
LOC787476	1.22	6.85	2.49	5.00E-05	1.05E-03
MAGED1	38.33	134.43	1.81	5.00E-05	1.05E-03
MAGIX	4.46	13.29	1.57	2.80E-03	2.53E-02
MBNL3	23.27	45.11	0.95	1.46E-02	8.20E-02
MCTS1	4.23	14.26	1.75	1.30E-02	7.59E-02
MECP2	3.08	5.49	0.83	6.90E-03	4.86E-02
MED12	34.31	91.25	1.41	5.00E-05	1.05E-03
MED14	17.32	31.84	0.88	9.50E-04	1.12E-02
MORF4L2	12.60	29.20	1.21	2.50E-04	3.94E-03
MPP1	12.42	42.61	1.78	5.00E-05	1.05E-03
MXRA5	9.22	21.47	1.22	5.00E-05	1.05E-03
NDUFB11	168.27	258.60	0.62	3.51E-02	1.49E-01
NONO	218.33	613.16	1.49	5.00E-05	1.05E-03
NSDHL	10.02	27.72	1.47	3.00E-04	4.56E-03
OCRL	66.66	107.05	0.68	4.55E-03	3.61E-02
OGT	65.84	178.02	1.43	5.00E-05	1.05E-03
OTUD5	28.24	73.14	1.37	5.00E-05	1.05E-03
PASD1	0.15	4.41	4.89	1.89E-02	9.81E-02

PDK3	21.92	42.08	0.94	3.00E-03	2.67E-02
PDZD11	80.51	32.78	-1.30	2.00E-04	3.30E-03
PGK1	26.04	15.49	-0.75	3.14E-02	1.38E-01
PHF16	2.98	6.87	1.21	1.72E-02	9.20E-02
PHF8	52.46	162.53	1.63	5.00E-05	1.05E-03
PHKA2	1.12	4.16	1.89	1.04E-02	6.50E-02
PIM2	2.64	5.52	1.07	3.09E-02	1.37E-01
POLA1	15.15	26.06	0.78	5.30E-03	4.02E-02
PORCN	2.73	9.54	1.81	3.50E-04	5.15E-03
PRAF2	3.23	17.84	2.47	1.10E-03	1.26E-02
PRICKLE3	1.70	11.17	2.71	3.50E-04	5.15E-03
PSMD10	36.95	74.38	1.01	4.15E-03	3.38E-02
RAB39B	1.16	4.19	1.85	5.55E-03	4.15E-02
RBM10	48.89	130.45	1.42	5.00E-05	1.05E-03
RBMX2	13.44	24.67	0.88	2.47E-02	1.18E-01
RBMX2	15.15	42.57	1.49	1.60E-03	1.68E-02
RLIM	77.16	50.54	-0.61	1.19E-02	7.12E-02
RPL10	386.00	225.37	-0.78	4.70E-03	3.69E-02
RPS4X	812.36	1863.69	1.20	5.00E-05	1.05E-03
RRAGB	0.10	2.06	4.30	3.36E-02	1.45E-01
SCML2	0.10	2.63	4.72	4.35E-02	1.72E-01
SFRS17A	0.17	1.62	3.28	3.55E-02	1.50E-01
SH3KBP1	28.80	44.06	0.61	2.94E-02	1.32E-01
SLC10A3	7.46	13.54	0.86	2.69E-02	1.25E-01
SLC16A2	17.62	30.39	0.79	5.70E-03	4.24E-02
SLC25A5	234.75	352.39	0.59	2.34E-02	1.13E-01
SLC6A8	89.23	299.96	1.75	5.00E-05	1.05E-03
SLC9A6	5.44	9.36	0.78	2.40E-02	1.16E-01
SLITRK2	13.28	50.31	1.92	5.00E-05	1.05E-03
SMC1A	149.16	242.05	0.70	3.85E-03	3.20E-02
SMS	70.32	42.94	-0.71	1.26E-02	7.43E-02
SRPX2	1.92	5.04	1.40	3.83E-02	1.58E-01
STAG2	27.11	42.60	0.65	8.95E-03	5.85E-02
STS	1.16	3.35	1.53	1.79E-02	9.43E-02
SUV39H1	4.53	10.74	1.24	6.00E-03	4.40E-02
TAF1	11.96	19.09	0.68	1.56E-02	8.59E-02
TBC1D25	3.50	9.51	1.44	8.10E-03	5.45E-02
TFE3	27.23	55.51	1.03	5.00E-05	1.05E-03
TIMM17B	10.86	28.45	1.39	7.30E-03	5.06E-02
TIMP1	20.21	114.60	2.50	5.00E-05	1.05E-03
TM9SF2	4.71	1.49	-1.66	1.90E-02	9.83E-02
TMEM35	0.09	1.55	4.04	4.33E-02	1.72E-01
TSPYL2	13.31	23.95	0.85	7.50E-03	5.16E-02
TXLNG	9.56	17.68	0.89	2.11E-02	1.06E-01
UBA1	119.11	296.01	1.31	5.00E-05	1.05E-03
UBQLN2	6.40	26.03	2.02	5.00E-05	1.05E-03
UPRT	5.91	20.37	1.79	7.00E-04	8.87E-03
USP9X	18.86	27.71	0.55	3.40E-02	1.46E-01
USP9Y	10.78	0.00	-Inf	5.00E-05	1.05E-03
USP9Y	6.88	0.00	-Inf	5.00E-05	1.05E-03
USP9Y	1.41	0.00	-Inf	5.00E-05	1.05E-03
WDR13	1.50	5.43	1.86	7.15E-03	4.98E-02
XIAP	48.84	24.52	-0.99	1.40E-03	1.51E-02
XIST	15.34	158.51	3.37	5.00E-05	1.05E-03
XKRX	0.29	2.55	3.12	3.95E-02	1.62E-01
XPNPEP2	166.18	431.15	1.38	5.00E-05	1.05E-03
YIPF6	7.20	4.20	-0.78	4.27E-02	1.70E-01
ZBTB33	3.10	7.53	1.28	5.50E-04	7.35E-03
ZDHHC9	52.71	79.57	0.59	1.54E-02	8.53E-02

ZIC3	2.09	5.75	1.46	2.17E-02	1.08E-01
ZMYM3	26.40	58.53	1.15	5.00E-05	1.05E-03
ZNF449	2.09	6.35	1.60	1.99E-02	1.02E-01
