# Supplementary material for: Characterization of X-Chromosome Gene Expression in Bovine Blastocysts Derived by In vitro Fertilization and Somatic Cell Nuclear Transfer
Source: Front Genet. 2017 Apr 10;8:42. doi: 10.3389/fgene.2017.00042 (PMC5385346; doi:10.3389/fgene.2017.00042)
Supplement: Supplementary Table S3 — Sequencing depth and mapping rates. [file Table3.PDF]

Table S3. Sequencing depth and mapping rates

Males

Sample	Raw reads	Mapped reads	% mapped
IVF-1	20,357,850	19,420,425	95.40%
IVF-2	20,714,651	19,576,771	94.50%
IVF-3	17,832,574	16,826,374	94.40%
IVF-4	20,918,004	19,898,758	95.10%
IVF-5	17,457,199	16,655,035	95.40%
IVF-6	19,694,384	18,706,823	95.00%
NT-1	22,944,210	21,976,276	95.80%
NT-2	18,813,008	18,010,158	95.70%
NT-3	23,943,315	22,847,048	95.40%
NT-4	16,160,699	15,402,626	95.30%
NT-5	26,300,796	24,744,214	94.10%
NT-6	21,428,970	20,529,990	95.80%
Sham-1	22,435,496	11,655,219	51.90%
Sham-2	19,717,461	18,326,417	92.90%
Sham-3	31,043,123	16,376,388	52.80%
Sham-4	26,820,232	14,160,121	52.80%
Sham-5	27,620,472	17,044,641	61.70%
Sham-6	30,547,173	18,484,816	60.50%

Females

Sample	Raw reads	Mapped reads	% mapped
IVF-1	25,756,219	20,979,677	81.50%
IVF-2	21,182,984	17,200,292	81.20%
IVF-3	20,136,258	16,403,987	81.50%
IVF-4	20,294,677	17,087,734	84.20%
IVF-5	21,250,344	17,256,794	81.20%
IVF-6	30,633,844	25,572,094	83.50%
NT-1	31,089,033	26,483,180	85.20%
NT-2	26,717,996	22,255,056	83.30%
NT-3	27,993,372	23,490,906	83.90%
NT-4	31,269,350	26,051,171	83.30%
NT-5	14,777,987	11,670,598	79.00%
NT-6	17,839,949	14,864,269	83.30%
Sham-1	15,559,075	12,916,328	83.00%
Sham-2	17,365,511	14,215,401	81.90%
Sham-3	18,314,372	15,624,999	85.30%
Sham-4	16,482,058	13,909,142	84.40%
Sham-5	16,347,416	13,732,432	84.00%

Donor cells

Sample	Raw reads	Mapped reads	% mapped
BESF_Female	25,400,027	20,112,949	79.20%
BESF_Male	26,046,124	22,058,329	84.70%
